# Research progress on osteoclast regulation by biodegradable magnesium and its mechanism

**DOI:** 10.1093/rb/rbaf026

**Published:** 2025-04-26

**Authors:** Wangwei Zhu, Weidan Wang, Xing Yang, Chunxiao Ran, Tianwei Zhang, Shibo Huang, Jiahui Yang, Fuyang Wang, Huiya Wang, Peng Wan, Fengyuan Piao, Faqiang Lu, Shengbo Shi, Ye Li, Xiuzhi Zhang, Dewei Zhao

**Affiliations:** Department of Orthopedics, Affiliated Zhongshan Hospital of Dalian University, Dalian 116001, China; Orthopedic Medical Research Center, Dalian University, Dalian 116622, China; Institute of Metal Research, Chinese Academy of Sciences, Shenyang 110016, China; Department of Orthopedics, Affiliated Zhongshan Hospital of Dalian University, Dalian 116001, China; Department of Orthopedics, Affiliated Zhongshan Hospital of Dalian University, Dalian 116001, China; Department of Orthopedics, Affiliated Zhongshan Hospital of Dalian University, Dalian 116001, China; Department of Orthopedics, Affiliated Zhongshan Hospital of Dalian University, Dalian 116001, China; Department of Orthopedics, Affiliated Zhongshan Hospital of Dalian University, Dalian 116001, China; Department of Orthopedics, Affiliated Zhongshan Hospital of Dalian University, Dalian 116001, China; Department of Orthopedics, Affiliated Zhongshan Hospital of Dalian University, Dalian 116001, China; School of Materials Science and Engineering, Dongguan University of Technology, Dongguan 523808, China; Department of Orthopedics, Affiliated Zhongshan Hospital of Dalian University, Dalian 116001, China; Department of Orthopedics, Affiliated Zhongshan Hospital of Dalian University, Dalian 116001, China; Department of Orthopedics, Affiliated Zhongshan Hospital of Dalian University, Dalian 116001, China; Department of Rehabilitation Sciences, The Hong Kong Polytechnic University, Hong Kong SAR 999077, China; Orthopedic Medical Research Center, Dalian University, Dalian 116622, China; Department of Orthopedics, Affiliated Zhongshan Hospital of Dalian University, Dalian 116001, China

**Keywords:** biodegradable magnesium alloys, osteoclast, osteoblast, regulatory mechanisms, bone remodelling

## Abstract

Continuous advancements in medical technology and biomaterials have underscored the significant advantages of biodegradable implant materials for bone repair and remodelling over traditional inert metallic implants. Notably, biodegradable magnesium-based materials have gained much attention because of their optimal corrosion rates. Importantly, extensive clinical experience has resulted in the use of biodegradable magnesium-based orthopaedic implants. Both preclinical and clinical studies have consistently demonstrated that Mg has an excellent ability to promote bone tissue formation, a process that is closely associated with the release of Mg^2+^ and other degradation byproducts. Bone metabolism depends on a dynamic balance of bone formation and bone resorption. Mg^2+^ has been shown to increase osteoblast (OB) activity while suppressing osteoclast (OC) formation, thus playing a crucial role in bone remodelling and regeneration. In terms of osteolysis inhibition, Mg^2+^ plays a multifaceted role. First, Mg^2+^ inhibits OC formation by modulating the activity of mature OCs, their migratory behaviour and the activity of precursor cells. Second, Mg^2+^ influences OC production by regulating the expression of osteoprotegerin (OPG), receptor activator of nuclear factor kappa-Β ligand (RANKL) and nuclear factor kappa-light-chain-enhancer of activated B cells (NF-κB). Additionally, Mg^2+^ impacts bone resorption by altering the immune microenvironment and the levels of hormones and peptides within the body. Furthermore, the alkaline environment generated around the biodegradable magnesium implant and its degradation products (e.g. H_2_) also significantly inhibit OC formation. Recent research on magnesium-based implants has focused predominantly on their osteogenic properties, with few systematic reviews addressing the mechanisms through which biodegradable magnesium alloys suppress osteoclastic activity. This article summarizes the latest clinical research progress concerning biodegradable magnesium implant materials and their significant regulatory effects and discusses recent advances in the understanding of the regulatory mechanisms of action Mg-based biomaterials on OCs, with the aim of providing a more theoretical basis for the clinical application of biodegradable magnesium-based implants.

## Graphical abstract

**Figure:**
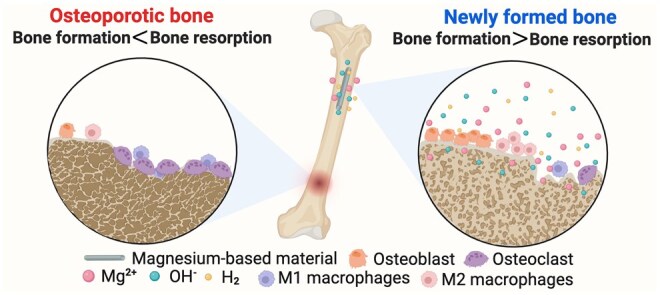

## Introduction

Bone formation is a highly dynamic and finely regulated physiological process involving complex interactions between osteoblasts (OBs) and osteoclasts (OCs). OCs provide space for new bone formation by absorbing the ageing bone matrix, whereas OBs synthesize and mineralize the new bone matrix in these spaces, thereby promoting bone tissue remodelling and maintaining bone structural integrity. This process depends on the balance and coordination between OBs and OCs. When OC activity is abnormally enhanced or OB function is weakened, bone mineral density may decrease, which may lead to the occurrence of bone metabolic diseases such as osteoporosis.

**Figure 1.**
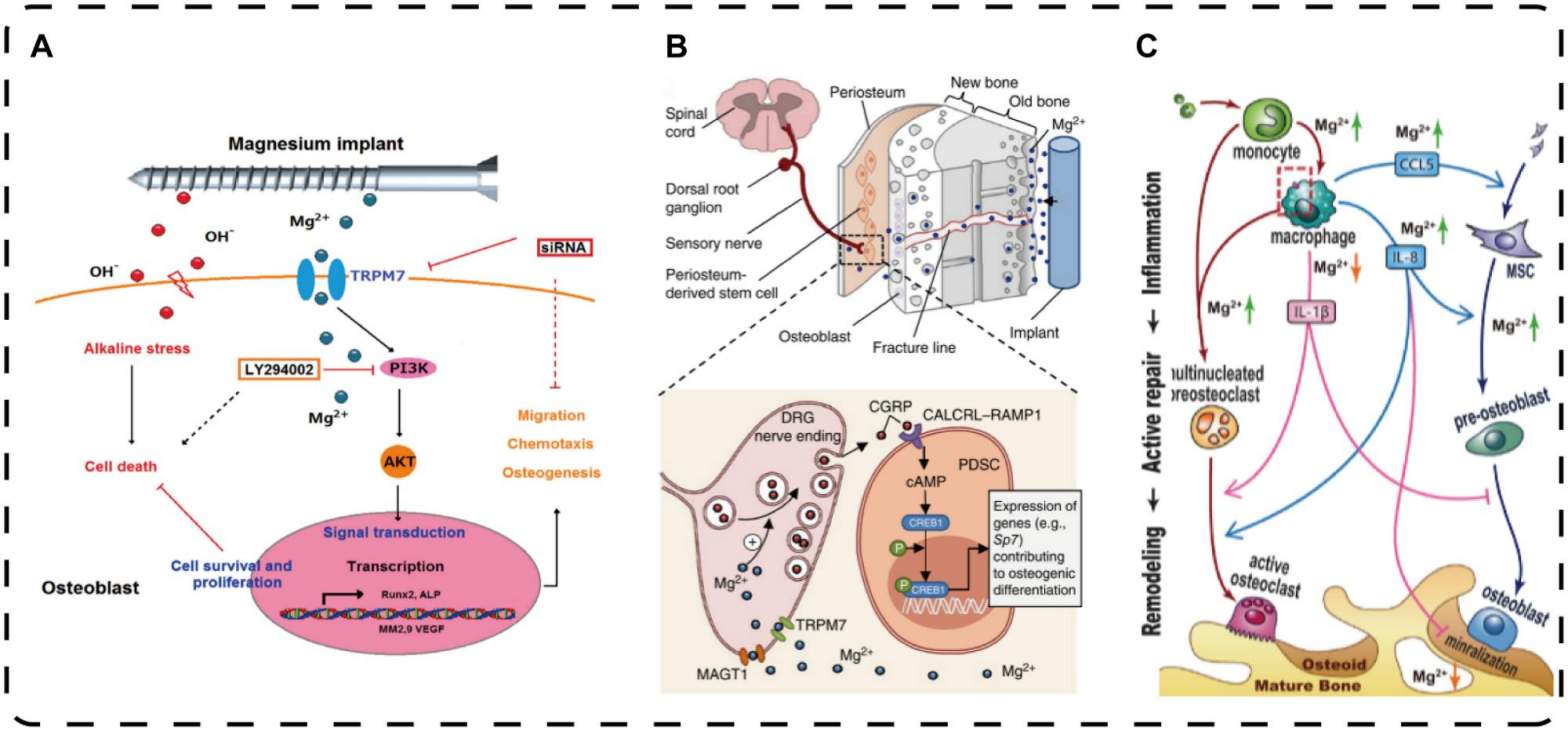
Mechanism by which magnesium promotes osteogenesis. (**A**) Schematic showing how the TRPM7/PI3K signalling pathway mediated by magnesium ions promotes osteogenesis and resistance to alkaline stress-induced cytotoxicity in human osteoblasts. Adapted and reproduced from Ref. [1] Copyright © 2017 Acta Materialia Inc. Published by Elsevier Ltd All rights reserved. (**B**) Schematics showing the release of Mg^2+^ through Mg^2+^ transporters or channels (i.e. MAGT 1 and TRPM 7) and the promotion of CGRP-vesicle accumulation and exocytosis. The CGRP released by DRGs in turn activates the CGRP receptor (composed of CALCRL and RAMP 1) in PDLSCs, which triggers the phosphorylation of CREB 1 via cAMP and promotes the expression of genes that contribute to osteogenic differentiation. Adapted and reproduced from Ref. [2]. (**C**) Schematic showing the mechanism by which Mg^2+^ regulates macrophages and mesenchymal stem cells during bone healing. Figure is licenced under CC by-NC-ND 4.0 [3]. Copyright © 2021, the Author(s).

With increasing research on bone health issues, the potential of biodegradable magnesium in bone repair and regeneration has begun to receive increased attention. As a biocompatible material with good mechanical properties, biodegradable magnesium has shown great application prospects in bone tissue engineering. Unlike traditional metal materials, the degradation process of magnesium in the body can be coordinated with the regeneration process of bone but does not cause long-term metal residue problems. This property makes magnesium an ideal bone repair material, improving bone metabolism by regulating the interaction of OBs and OCs and providing effective support for bone health. When the magnesium material is degraded, the released magnesium ions can mediate the TRPM7/PI3K signalling pathway and other mechanisms to promote osteogenesis (Figure 1A–C) [1–3]. Moreover, by inhibiting NF-κB activity, the expression of NFATc1 decreases, affecting the expression of TRAP, MMP-9 and cathepsin K, which prevents excessive OCs activity and slows bone resorption, effectively promoting bone repair and regeneration.

Owing to the excellent properties of biodegradable magnesium-based materials, they have been preliminarily applied in clinical practice for fracture fixation and bone defect repair. Additionally, significant progress has been achieved in clinical research on magnesium alloys. For example, the Mg–Y–Re–Zr alloy screw Magnezix, manufactured by Syntellix AG in Germany, is the first in the world to be used in orthopaedic surgery for bunions; the K-Met screw with a Mg–5 wt% Ca–1 wt% Zn alloy developed by the U&I Company of Korea is used for the treatment of distal radius fracture repair [4, 5] and the 99.99% high-purity magnesium screw developed by the Chinese research team is used for the clinical fixation of autogenous vascular bone flaps in the treatment of avascular necrosis of the femoral head [6], achieving satisfactory outcomes (Table 1).

**Table 1.** Clinical research status of magnesium alloys

Mg-based metals	Time	Surgical site	Country	Number of cases	Postoperative follow-up time (month)	Osteogenesis or fracture healing	Ref.
Mg alloy (Mg–Y–RE–Zr)	2010–2011	Correction of hallux valgus	Germany	13	36	Complete healing	[4]
Pure Mg	2012–2013	Displaced femoral neck fracture in the young	China	16	19	No avascular necrosis of the femoral head occurred	[7]
Mg alloy (Mg–5wt%Ca–1wt%Zn)	2013–2014	Internal fixation of metacarpal and carpal fractures	South Korea	53	12	Complete healing	[5]
Mg alloy (Mg–Y–RE–Zr)	2013–2015	Hallux valgus correction	Germany	40	6	90% healing after 12 weeks	[8]
Pure Mg	2013–2015	Graft band for ischaemic necrosis of the femoral head vascular bone flap fixation	China	12	25	No displacement or collapse of bone flap after surgery	[6]
Mg alloy (Mg–Y–RE–Zr)	2015–2016	Internal fixation of internal ankle fractures	Turkey	11	12	All fractures healed	[9]
Mg alloy (Mg–Y–RE–Zr)	2015–2018	Internal fixation of internal ankle fractures	Turkey	11	12–24	All fractures healed	[10]
Mg alloy (Mg–Nd–Zn–Zr)	2018–2019	Internal fixation of internal ankle fractures	China	9	12.2	All fractures healed	[11]
Mg alloy (Mg–Zn–Ca)	2018–2019	Medial malleolar fractures fixation	Austria	20	18–43	Full resorption and good ankle function	[12–14]

Bone remodelling is a dynamic repair process in which bone tissue is continuously metabolized and rebuilt. Osteogenesis and OC activity are two basic processes in bone remodelling and are closely linked and carried out in dynamic equilibrium [15]. Inflammatory factors and cytokines are the key molecules that regulate the dynamic balance of osteogenesis and OC activity and realize bone remodelling [16]. The abnormal expression of inflammatory factors and cytokines is an important cause of abnormal bone remodelling. The oversecretion of inflammatory factors *in vivo*, including white blood cells IL-1, IL-6 and IL-11, TNF-α and IFN-γ, is an important reason for the overactivation of OCs [17–19]. Many cytokines, such as FGF, TGF-β, M-CSF and RANKL, can also lead to the overactivation of OCs [20–23]. Most bone metabolic diseases, such as osteoporosis, periodontitis, degenerative arthritis and Paget's disease, are caused by the abnormal activity of OCs [24–27]. Therefore, biomaterials for the repair of bone metabolic diseases caused by OC overactivity should have the dual functions of promoting osteogenesis and inhibiting OC activity. Mg-based biodegradable materials strongly inhibit bone resorption and regulate the immune response. A number of studies have shown that magnesium-based materials, such as NT-Mg and pure Mg materials, can reduce bone resorption by inhibiting OC formation or activity by inhibiting the NF-κB and NFATc1 signalling pathways to play an antiresorption role [28]. In addition, Mg-based materials can promote the polarization of M2-type macrophages, inhibit M1-type macrophages and regulate the immune response, thus further supporting the bone repair process (Table 2). These reports suggest that magnesium-based materials have important potential applications in bone repair and immune regulation. At present, the design of bone tissue engineering implant materials has changed from ‘immune-friendly’ to ‘immune-modulating’. Among them, macrophages are the regulatory centre of the immune response process and play a key role in bone tissue repair and regeneration. The results of a study by Yeung *et al.* [3] revealed that an increase in the magnesium ion concentration significantly increases the concentration of magnesium ions in macrophages and upregulates the expression of TRPM7, thus promoting the recruitment of mononuclear macrophages and the development of an immune microenvironment for bone tissue regeneration by promoting M2-type polarization. In bone metabolism, the presence of M1-type macrophages leads to the intensification of OC activity, whereas M2-type macrophages help inhibit bone resorption processes. Studies have shown that M1 macrophages can secrete a variety of proteases and cytokines, such as MMPs and RANKL [37, 38]. These substances can promote OCs to secrete enzymes that degrade the bone matrix, thus accelerating OC activity. In contrast, M2-type macrophages can secrete IGF-1 and TGF-β, which help promote bone cell secretion from the bone matrix and increase bone density, which is conducive to inhibiting the bone resorption process [39, 40]. Therefore, biodegradable magnesium can actively play an immunomodulatory role in tissue engineering repair through the release of magnesium ions and has important significance in regulating the dynamic balance of bone regeneration and bone resorption during bone remodelling.

**Table 2.** Research on biodegradable Mg-based implants

Biodegradable Mg-based materials	Cells	Animals	Impact of materials on OC	Related mechanisms	Refs.
NT-Mg	hBMSCs	Sprague‒Dawley rats	Inhibition of osteoclast formation	Inhibition of NF-κB activity and NFATc1 expression	[28]
Mg-ALg	THP1 hBMSCs BMMs	Sprague‒Dawley rats	Early inhibition and late activation of osteoclast formation	Early promotion of macrophage surface CD206 and CD136 expression while downregulating OSM, IL-6, IL-1β, TNF-α expression, etc. Late-stage leads to excessive activation of NF-κB in macrophages	[3]
MgO&SA@PLGA microspheres	BMSCs	Sprague‒Dawley rats	Inhibition of osteoclast formation Decreased bone resorption activity	Inhibition of Akt phosphorylation	[29]
Biosynthesized bandages carrying MgONps	Macrophages BMSCs	Sprague‒Dawley rats	The number of osteoclasts did not increase	During osteoclast induction, the number of osteoclasts and the transcription levels of matrix degradation-related genes Nfatct1, Mmp9, Atp6v0d2 and Ctsk did not increase Significantly upregulated transcription levels of anti-inflammatory and M2 polarization-related genes	[30]
GeLMA-BP-Mg	BMSCs HUVECs	Sprague‒Dawley rats	Inhibition of osteoclast formation	Not mentioned in the text	[31]
Mg–*x*Si–*y*Ca	THP1-derived macrophages	Sprague‒Dawley rats	Significant decrease in osteoclast activity Marked reduction in bone resorption	Inhibition of IL-6 expression	[32]
Pure Mg	RAW264.7 BMMs	C57BL/6J mice	Inhibition of osteoclast formation	Inhibition of NF-κB inhibitory subunit (IκBα) phosphorylation and degradation, simultaneously affecting NFATc1 and its downstream genes MMP9, CTR and TRAP expression	[33]
PWH scaffold	RAW264.7 BMSCs	Sprague‒Dawley rats	Inhibition of osteoclast activation	Inhibition of the expression of TRAP, MMP-9 and tissue protease K	[34]
PLGA/MgO-alendronate microsphere	RAW264.7	Sprague‒Dawley rats	Inhibition of osteoclast formation	Induces a higher proportion of Arg 1-positive cells (M2 macrophages) and a lower proportion of iNOS-positive cells (M1 macrophages) at the site of injury	[35]
MMSC	RAW264.7 rbMSCs	Sprague‒Dawley rats	Inhibition of osteoclast formation	Promotes M2 phenotypic polarization and the expression of anti-inflammatory cytokines in RAW 264.7 macrophages Inhibits the formation of M1 phenotype macrophages	[36]

Although many studies have focused on the application of biodegradable magnesium-based materials in tissue engineering and biomedicine in recent years, especially their bone-promoting effect on OBs, the effects of magnesium-based materials on OCs are still insufficient. The key role of OCs in bone metabolism has been widely recognized, and their function not only affects the bone resorption process but is also closely related to the overall homeostasis of bone [41]. Most existing studies have focused only on the positive regulatory effects of magnesium on OBs, but the effects and regulatory mechanisms of magnesium on OCs in a dynamic degradation environment are not well understood. Recent studies have shown that magnesium not only plays a role in promoting osteogenesis but also regulates the function of OCs through complex signalling pathways, showing bidirectional regulatory characteristics. This review aims to fill this research gap and systematically explore how substances such as Mg^2+^ and hydrogen released during the degradation of magnesium-based materials regulate the biological behaviour of OCs in a dynamic environment, especially how they indirectly regulate OC differentiation by affecting OC signalling pathways and macrophage polarization [31, 42, 43]. In addition, exploring the ‘material–immune–bone metabolism’ cross-mechanism of the interaction between magnesium-based materials and the immune system provides a new theoretical perspective for the development of magnesium-based medical materials. This review not only enriches the biological function theory of magnesium-based materials but also provides more detailed guidance for their clinical application, which has important theoretical and practical application value.

## Effects of Mg on OCs and their precursors

**Figure 2.**
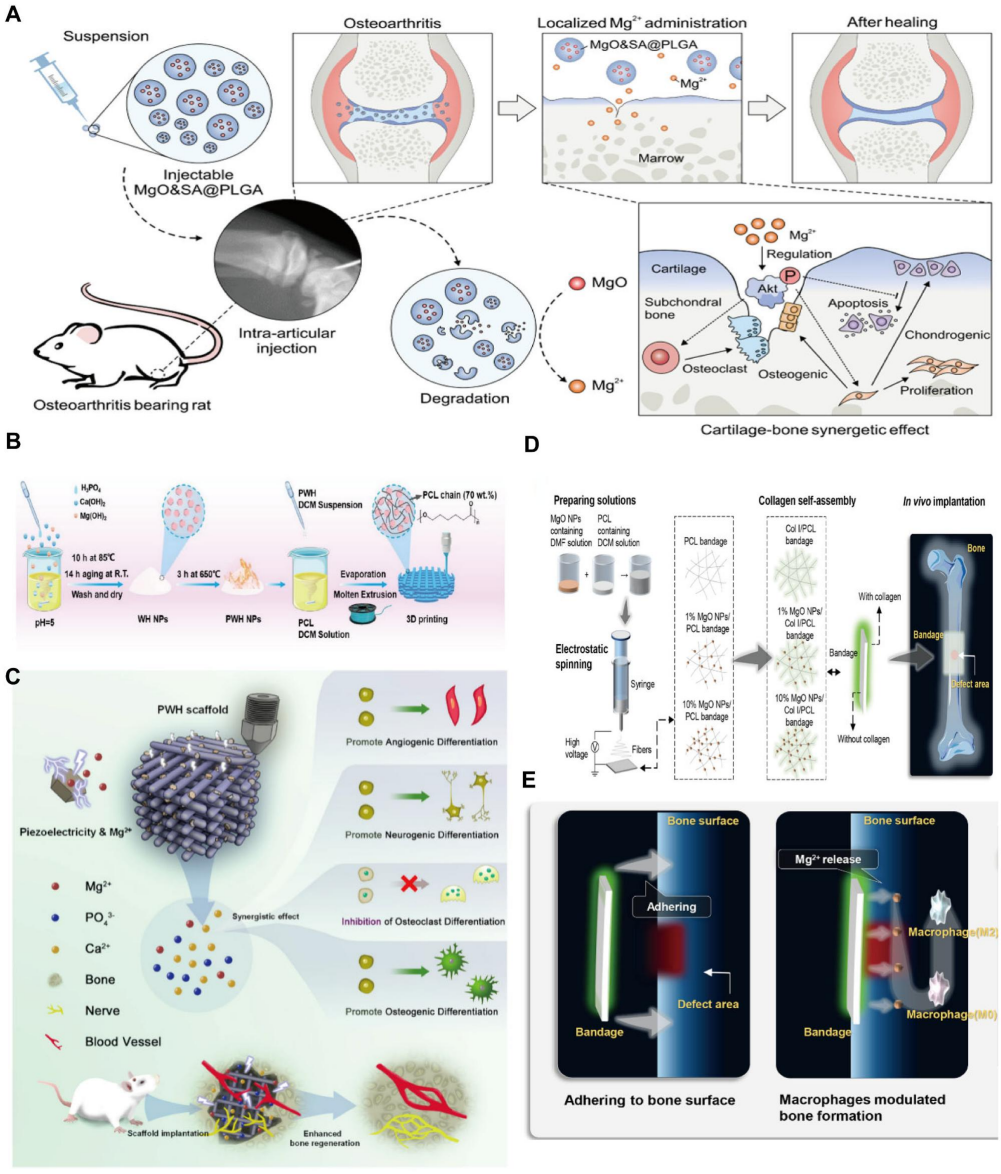
Preparation process of magnesium-based biomaterials and their mechanism of action on osteoclasts. (**A**) The continuous release of Mg^2+^ inhibits osteoclast formation by regulating AKT phosphorylation through intra-articular injection of MgO&SA@PlGA. Figure is licenced under CC by-NC-ND 4.0 [29]. Copyright © 2024, the American Association for the Advancement of Science. (**B**) For the preparation process of a composite scaffold composed of piezoelectric WH (PWH) and poly(ε-caprolactone) (PCL). (**C**) Schematic diagram showing the PWH scaffold prepared from WH NPs using 3D printing and PCL/PWH composite filaments. It inhibits osteoclast differentiation through the continuous and stable release of Mg^2+^. It also promotes the formation of nerves and blood vessels. Figure is licenced under CC by-NC-ND 4.0 [34]. Copyright © 2022 the Authors. Publishing services by Elsevier B.V. on behalf of KeAi Communications Co. Ltd. (**D**) Collagen is assembled on the surface of an electrospun fibre membrane coated with MgO nanoparticles to form a magnesium-based artificial bandage structure. (**E**) A magnesium-based artificial bandage attached to the surface of a mouse femur promotes the differentiation of macrophages into the M2 phenotype by releasing Mg^2+^. Adapted and reproduced from Ref. [30] Copyright © 2022, American Chemical Society.

As a biodegradable bone implant material, magnesium can not only promote bone activity but also inhibit osteoclastic activity in various ways, especially in regulating the activity of OCs and their precursor cells. OCs are the only cells responsible for bone resorption, and changes in their activity are closely related to diseases such as osteoporosis (Figure 2). Studies have shown that magnesium can affect not only the adhesion of OCs but also the proliferation and differentiation of precursor cells to slow bone loss. The release of magnesium ions during the degradation of magnesium-based biomaterials can lead to the formation of a local alkaline environment, which may be an important factor in inhibiting the activity of OCs, reducing the adhesion of OCs and reducing the differentiation of precursor cells into OCs (Figure 2) [44].

### Mg can inhibit the activity of OCs

**Figure 3.**
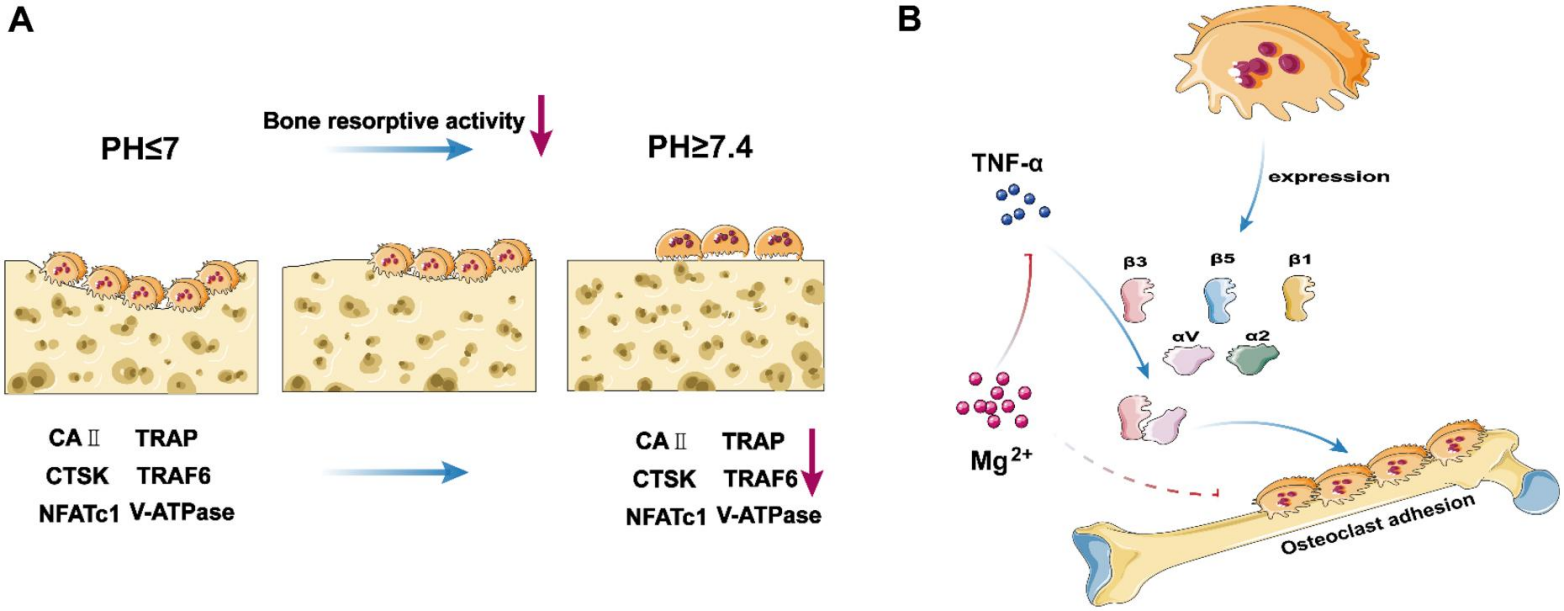
(**A**) The alkaline environment generated by the degradation of magnesium-based materials has been found to partially inhibit the expression of factors associated with osteoclast absorption and the bone resorption activity of osteoclasts. (**B**) Schematic diagram showing osteoclasts capable of α2, αV, β1, β3 and β5. By increasing the expression of αV and β3, TNF-α can enhance the adhesion ability of osteoclasts. Mg^2+^ inhibits bone resorption by inhibiting TNF-α.

Biodegradable magnesium-based materials have a potential inhibitory effect on OC activity. OC-mediated bone resorption activity is pH dependent [33]. In the case of acidosis, OC activity is significantly increased, whereas an alkaline environment inhibits OC activity [45, 46]. Therefore, the local alkaline environment caused by the degradation of Mg^2+^ released by biodegradable magnesium-based implants may be an important factor leading to the inhibition of OC activity. Studies have shown that OCs have little activity when the pH is 7.4, but even a small change with a gradual decrease in pH can lead to an increase in OC bone absorption activity. The study of Miana *et al.* again supported this view [44]. When the pH is reduced to approximately 7, OC activity can reach the best state [47–49]. Histamine (H1) plays a key role in the activation of OCs [48, 50]. Studies have shown that OC is highly sensitive to extracellular H1, which results in even a slight decrease in pH, leading to a doubling of bone resorption activity [51]. Therefore, the alkaline microenvironment generated by the degradation of magnesium-based implants around bone tissue may lead to relatively ideal inhibition of OC absorption. However, we still know very little about the mechanism involved. Studies have shown that the H1-sensitive G protein-coupled receptors TDAG8 and OGR1 expressed on OCs and the extracellular ATP PTX2 receptor expressed by OCs (activated by acid) are not involved in the regulation of OC activity [52, 53]. However, it is clear that acid activation is a critical initial requirement for OC formation, rapidly increasing the expression of carbonic anhydrase II and vacuolar H1-ATPase (required for proton production and the pumping of dissolved minerals) in OCs and strongly upregulating cathepsin K (required for organic matrix degradation) expression [54–56]. Moreover, the establishment of an acidic environment is conducive to the upregulation of the expression of bone resorption-related factors (TNF, NFATc1 and TRAP) [49] (Figure 3A). Therefore, the locally formed alkaline environment of Mg implant materials inhibits the expression of OC-related enzymes and factors, which may inhibit the activity of OCs.

### Mg can inhibit bone resorption by regulating the adhesion of OCs

OCs are highly polarized cells. When OCs are differentiated and mature, they establish a relatively closed microenvironment on the bone surface through adhesion, thus mediating the occurrence of bone resorption. Moreover, αVβ3 plays an important role in this process. At least two alpha-integrins (α2 and αV) and three β-integrins (β1, β3 and β5) are expressed in OCs [57]. Among them, αV and β3 regulate the adhesion of OCs on the bone surface to form a closed region by binding to the RGD sequence of bone matrix proteins (osteopontin, hyalonin, bone sialoprotein, etc), thereby regulating the bone resorption function. Studies have shown that TNF-α can enhance the adhesion ability of OCs by increasing the expression of αV and β3 [57, 58] (Figure 3B). Therefore, the release of TNF-α leads to the increased expression of αVβ3, which mediates bone resorption. Some studies have shown that Mg^2+^ can inhibit the formation and release of TNF-α to a certain extent. Therefore, biodegradable magnesium implants may affect the adhesion of OCs and inhibit the occurrence of bone resorption through the release of Mg^2+^ during degradation.

### Effects of Mg on the activity of OC precursors

OC differentiation from precursor mononuclear macrophages has been demonstrated. Therefore, some researchers believe that magnesium may also inhibit OC generation by inhibiting OC precursor cell activity. For example, Yang *et al.* [28] extracted titanium with a magnesium coating (NT-Mg1, NT-Mg3) and obtained extracts 48 h later (pH 9.1 and 8.7, respectively) for coculture with OCs. The results showed that the alkaline environment formed by the degradation of the magnesium coating inhibited the formation of rat bone marrow macrophages (rBMMs) into OCs [28]. Marina *et al.* [44] suggested that a lack of Mg^2+^ might lead to the active proliferation of OC precursor cells, leading to the production of more OCs. They analysed the changes in DNA content in long bone marrow cells with different Mg^2+^ concentrations through cell proliferation experiments, but the results revealed that there was no significant difference in DNA content under different Mg^2+^ concentrations. These findings indicate that the Mg^2+^ produced during magnesium alloy degradation may have no significant effect on the proliferation of OC precursor cells. However, owing to relatively few experimental reports in this regard, more experimental studies are needed to verify these findings. Diana *et al.* [59] reported that the metabolic activity of RAW267.4 cells in high-concentration Mg^2+^ medium was significantly reduced, and in the presence of Mg-conditioned medium, the ability of OC precursors to fuse into multinuclear OC-like cells was reduced, resulting in a large number of undifferentiated cells. These findings suggest that the decrease in the number of multicellular OC-like cells may be due to the inhibition of intercellular fusion. It has been reported that an acidic environment can indeed induce or trigger the fusion of macrophages, whereas an alkaline environment has the opposite effect [60]. Therefore, the decreased fusion ability of OC precursors may be caused by the alkaline environment generated by magnesium degradation. However, whether biodegradable Mg-based implant materials can affect the activity of OC precursor cells through other mechanisms remains to be further studied.

## Effect of Mg on the OPG/RANK/RANKL signalling pathway

**Figure 4.**
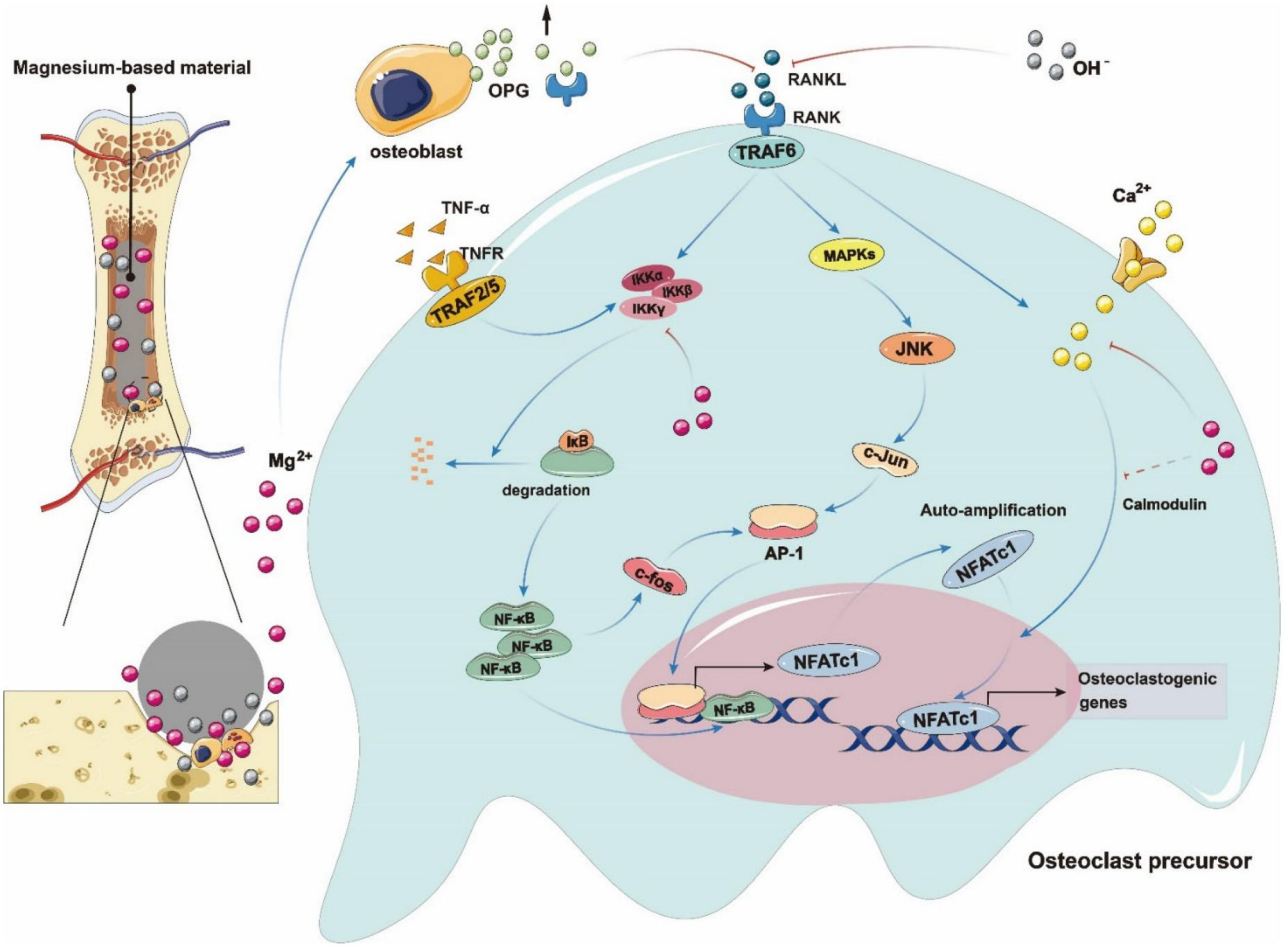
Biodegradable magnesium-based implants inhibit osteoclast differentiation through multiple mechanisms. First, magnesium ions (Mg^2+^) promote the binding of OPG secreted by osteoblasts to RANKL during material degradation, competitively inhibiting the binding of RANK to RANKL. Second, the alkaline environment generated by the degradation of magnesium-based materials further inhibits the binding of RANK and RANKL and reduces the formation of osteoclasts. In addition, magnesium ions inhibit the activation of inflammatory factors (such as TNF-α), affect the expression of downstream signalling molecules (such as JNK, ERK, NF-κB and AP-1), reduce the expression of NFATc1 and inhibit osteoclast differentiation. Finally, magnesium ions (Mg^2+^) significantly inhibit calcium ion (Ca^2+^) influx, thereby inhibiting osteoclast differentiation by inactivating the nuclear factor κB (NF-κB) and activated T nuclear factor c1 (NFATc1) signalling pathways.

The OPG/RANK/RANKL signalling pathway is crucial for regulating OCs [61, 62]. RANKL, which is essential for OC generation, is secreted by OBs and stromal cells and activates RANK, triggering downstream signalling molecules [61, 63]. NF-κB, a key downstream signalling molecule in the RANKL pathway, facilitates OC differentiation and maturation and mediates their formation by activating proinflammatory cytokines such as TNF-α [64]. Osteoprotegerin (OPG) serves as a soluble decoy receptor for RANKL, binding to it and thus preventing RANKL from interacting with RANK, which inhibits OC formation and bone resorption [65] (Figure 4).

### Effects of Mg on RANKL

**Figure 5.**
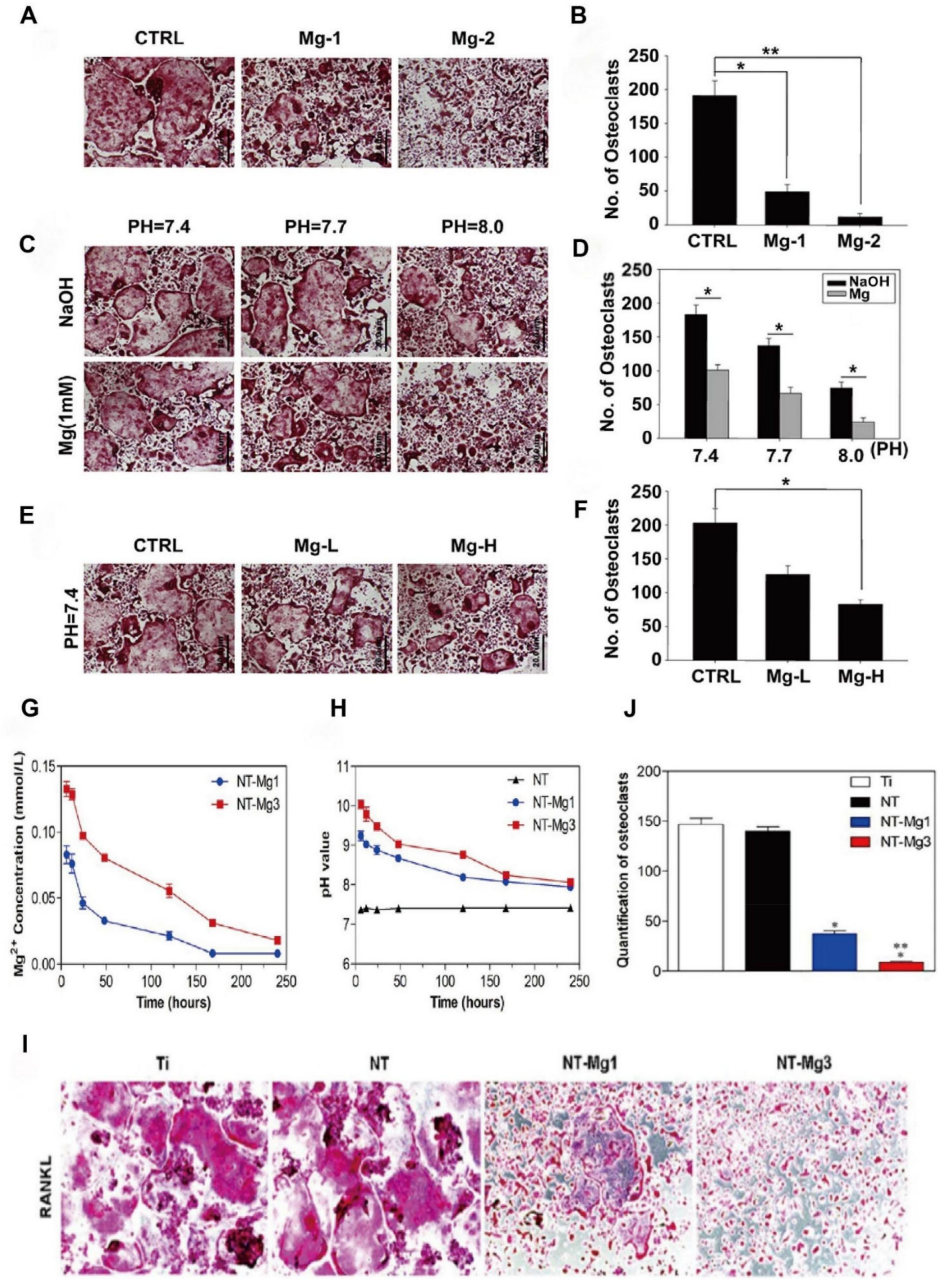
The effect of an alkaline microenvironment resulting from the degradation of magnesium-based materials on osteoclasts. (**A, B**) Inhibitory effect on osteoclasts, as determined by TRAP staining. (**C, D**) At different pH values (7.4, 7.7 and 8.0), the Mg (1 mm) group is more abundant than the NAOH group. (**E, F**) Inhibitory effect on osteocytes at the same pH (7.4). Adapted and reproduced from Ref. [33]. Copyright © 2014 Elsevier Ltd All rights reserved. (**G**) Changes in the Mg^2+^ concentration released during the degradation of NT-Mg1 and NT-Mg3 with time. (**H**) Relationships between the alkaline environments generated during the degradation of NT, NT-Mg1 and NT-Mg3 with time. (**I, J**) Inhibitory effects on osteoclasts, as determined by TRAP staining. Adapted and reproduced from Ref. [28]. Copyright © 2019, American Chemical Society.

RANKL can bind to the transmembrane protein RANK to promote the generation of OCs. It has been reported that RANKL can increase OC survival by modulating the mTOR (mammalian target of rapamycin)/S6K (ribosomal protein S6 kinase) and mTOR/Bim (proapoptotic members of the Bcl-2 protein family) pathways [66, 67]. Additionally, RANKL interacts with RANK on the surface of OCs and precursor cells, inducing RANK trimerization. This recruits TNF receptor-associated factor 6 (TRAF6) to bind with the cytoplasmic domain of the RANK receptor, activating downstream signalling molecules and thus promoting the differentiation and maturation of OCs [68, 69]. Magnesium can inhibit the expression of OC-related genes, especially RANKL, thereby reducing the formation of OCs and their bone resorption activity. Under magnesium-deficient conditions, RANKL treatment of rBMMs (rat bone marrow macrophages) can significantly stimulate the production of numerous TRAP-positive multinucleated OCs (Figure 5A and B). The expression of a series of OC-related genes (cathepsin K, CTR and NFATc1) is also significantly upregulated following RANKL stimulation [28]. Increasing the magnesium concentration, e.g. with the use of pure magnesium leachate (MLL), markedly inhibits the increase in NFATc1 induced by RANKL at both the mRNA and protein levels [33]. However, the mechanism by which magnesium-based implants affect RANKL is still poorly understood. Research has shown that acidosis can upregulate the expression of RANKL in bone tissue [70]. Komarova *et al.* [70], through immunofluorescence detection, reported that only approximately 30–40% of OCs accumulated NFATc1 in an alkaline environment (pH 7.6–7.9). As the pH gradually decreased, the number of OCs with NFATc1 accumulation increased, reaching a maximum (71 ± 3%) at a pH of 7.0. Zhai *et al.* [33] verified those findings, reporting that as the pH increased, the number of OCs also decreased (Figure 5C and D). This finding aligns with the conclusion that OC resorptive activity is mediated by pH. Therefore, the alkaline environment created by the degradation of magnesium-based implants may be an important factor in inhibiting RANKL and thus affecting the formation of OCs.

### Effects of Mg on NF-κB

**Figure 6.**
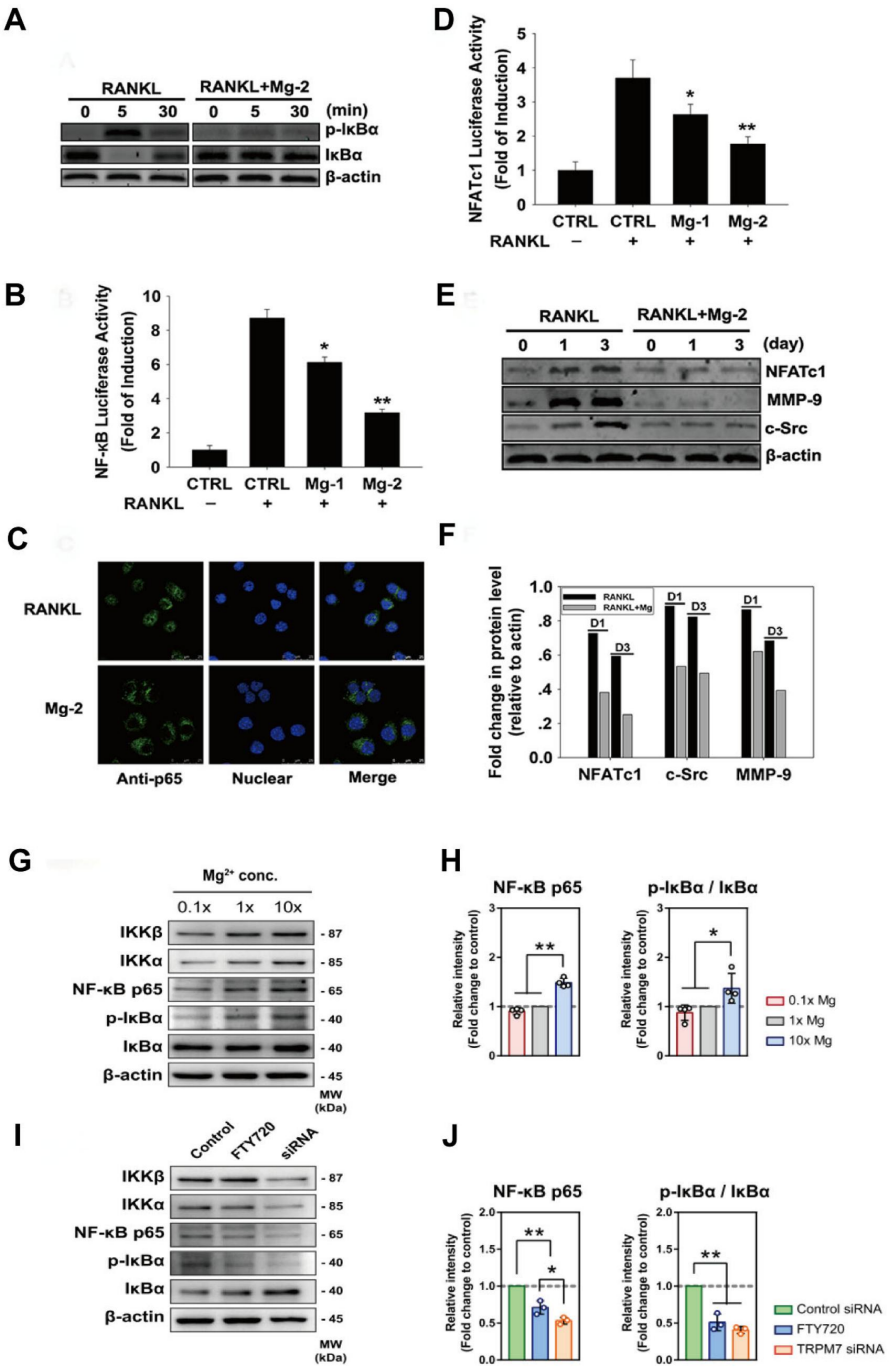
The effect of Mg^2+^ on genes related to osteoclast formation. (**A**) Western blot analysis confirmed that MLL inhibited the phosphorylation and degradation of the RANKL-induced NF-kB inhibitory subunit (IkBa). (**B**) The effect of MLL on NF-κB activity was assessed using luciferase gene assays. (**C**) The blockade of p65 nuclear transport. (**D**) The effect of MLL on NFATc1 activity was assessed using luciferase gene assays. (**E, F**) MLL inhibited the expression of genes related to osteoclast generation at both the mRNA and protein levels. Adapted and reproduced from Ref. [33] Copyright © 2014 Elsevier Ltd All rights reserved. (**G, H**) Representative Western blots and the corresponding quantification showing the concentration-dependent effect of Mg^2+^. Figure is licenced under CC by-NC-ND 4.0 [3]. Copyright © 2021, the Author(s). (**I, J**) The influence of FTY720 or TRPM7 siRNA on the activation ofNF-κB signalling in THP1-derived macrophages. Figure is licenced under CC by-NC-ND 4.0 [3]. Copyright © 2021, the Author(s).

Magnesium can influence the generation of OCs through multiple pathways affecting the NF-κB protein. On the one hand, magnesium can inhibit the phosphorylation and degradation of the NF-κB inhibitor protein IκB within OC precursor cells via the RANKL/RANK pathway, thereby preventing NF-κB from entering the nucleus to induce the activation of NFATc1, which is crucial for OC formation [71, 72]. For example, in related studies, magnesium was able to inhibit RANKL-induced phosphorylation and degradation of the NF-ĸB inhibitory subunit (IKBa) (Figure 6), and the nuclear translocation of the NF-ĸB subunit P65 was almost blocked [3, 28, 33] (Figure 6C, G and H). Magnesium is a natural calcium antagonist and a potent inhibitor of L-type calcium channels. Magnesium may inhibit the self-amplification of NFATc1 by inhibiting Ca^2+^-dependent calcineurin signalling. Recently, Zheng *et al.* [73] also showed that Mg^2+^ can significantly inhibit Ca^2+^ influx, thereby inhibiting OC differentiation by inactivating the NF-κB and NFATc1 signalling pathways. On the other hand, magnesium can inhibit the NF-κB pathway by suppressing the release of inflammatory cytokines such as TNF-α from macrophages [74]. Research has shown that magnesium depletion leads to elevated levels of TNF-α and substance P (a neuropeptide involved in local inflammatory responses), which in turn further increase TNF-α levels [75]. TNF-α then recruits TRAF2/5 to activate NF-κB, thereby promoting the expression of a series of genes related to OC formation [64]. Thus, magnesium plays a key role in the formation of OCs through the regulation of TNF-α. Previous studies have shown that magnesium primarily inhibits NF-κB. However, a recent study by Qiao *et al.* presented a different perspective [3]. In the later stages of bone remodelling, the prolonged administration of magnesium ions led to the excessive activation of NF-κB in macrophages, resulting in an increased number of OCs. These findings suggest that magnesium may promote bone formation only during the early stages of bone repair and that prolonged exposure to magnesium ions might have potential negative effects on bone healing. Consequently, this insight provides new ideas for the development of biodegradable magnesium implants: controlling the release rate of magnesium ions during degradation may more effectively promote bone formation.

### Effect of Mg on OPG

OPG, which is produced primarily by OBs, serves as a decoy receptor for RANKL; it competitively binds to RANKL and effectively inhibits OC formation [76]. Research shows that magnesium deficiency reduces OPG levels in bone tissue, thereby increasing OC activity. Increasing the dietary intake of magnesium-rich foods can increase serum OPG levels, supporting bone formation. Therefore, magnesium ions released from biodegradable magnesium implants could increase serum OPG levels, inhibit RANK binding to RANKL and suppress OC formation.

## Mg affects OCs by modulating the immune system

Chronic inflammatory responses can lead to excessive bone resorption, contributing to bone-related diseases such as osteoporosis. For example, rheumatoid arthritis is a common autoimmune disorder in which inflammatory reactions in the joints gradually destroy the surrounding bone tissue, ultimately leading to joint deformity and loss of function [77]. Mg^2+^ is an essential mineral in the human body and plays a crucial role in immune regulation. Research indicates that magnesium ions regulate bone resorption by modulating the bone immune microenvironment. Mg^2+^ has significant regulatory effects within the immune system, including modulating the polarization and function of macrophages. Macrophages are vital immune cells involved in phagocytosis and the secretion of various bioactive factors and play important roles in immune responses and tissue repair [78]. In bone metabolism, M1 macrophages increase OC activity, whereas M2 macrophages help inhibit bone resorption. Studies have shown that M1 macrophages secrete various proteases and cytokines, including matrix metalloproteinases (MMPs) and RANKL, which stimulate OCs to release enzymes that degrade the bone matrix, thereby accelerating bone resorption [79, 80]. In contrast, M2 macrophages secrete growth factors such as IGF-1 and TGF-β, which promote bone matrix formation, increase bone density and inhibit bone resorption [81]. Thus, biodegradable magnesium, through the release of magnesium ions, exerts important immunomodulatory effects in tissue engineering; it significantly influences the regulation of bone regeneration and bone resorption during the process of bone remodelling.

**Figure 7.**
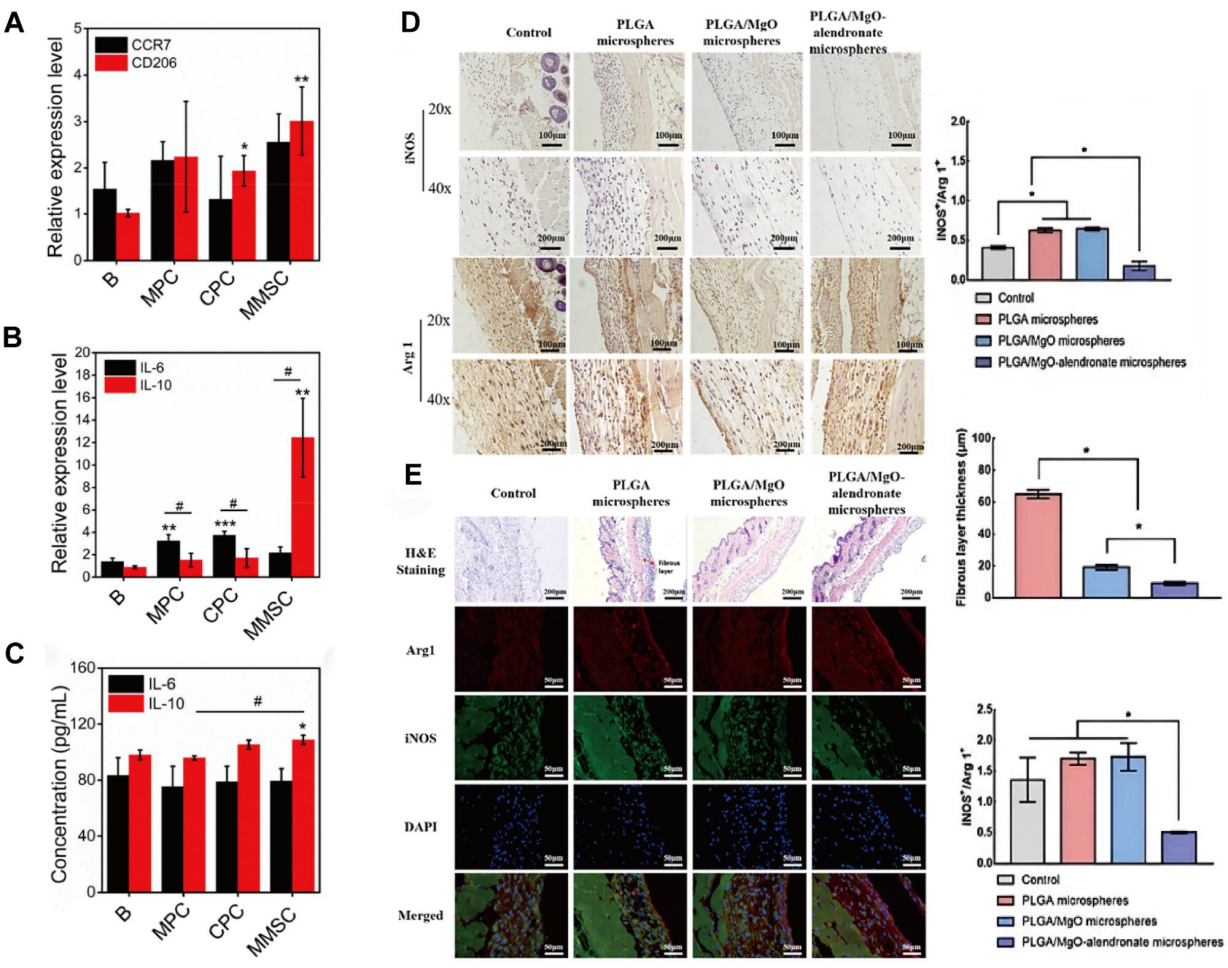
The mechanism by which Mg^2+^ affects immunomodulation *in vivo*. (**A**) Compared with the other groups, the bone cement (MMSC) group containing magnesium microspheres presented higher CD206 and CCR7 expression. (**B**) Compared with the other groups, the MMSC group containing magnesium microspheres presented higher IL-10 expression and lower IL-6 expression. (**C**) The IL-10 concentration in the MMSC group was significantly greater than that in the B and MPC groups. Figure is licenced under CC by-NC-ND 4.0 [36]. Copyright © 2021 the Authors. Publishing services by Elsevier B.V. on behalf of KeAi Communications Co. Ltd. (**D**) Quantitative and qualitative analyses of immunofluorescence and HE staining between the PLGA/MgO-alendronate sodium group and other groups. (**E**) Results of the quantitative and qualitative analyses of iNOS (green, M1 phenotype) and Arg 1 (red, M2 phenotype) expression via immunofluorescence and HE staining in 4D postoperative skin tissue sections. Figure is licenced under CC by-NC-ND 4.0 [35]. Copyright © 2021 the Authors. Production and hosting by Elsevier B.V. on behalf of KeAi Communications Co., Ltd.

First, Mg^2+^ can regulate the expression and activation of surface receptors and signalling pathways in macrophages. Recent studies, such as those by Zheng and colleagues on glucocorticoid-induced osteonecrosis, have shown that Mg^2+^ reduces inflammation by modulating intracellular calcium signalling pathways, which further inhibits OC formation, thereby alleviating osteonecrosis and promoting bone repair [73]. Additionally, Mg^2+^ can promote the expression of the CD206 receptor on the surface of macrophages, increasing the number and activity of M2 macrophages (Figure 7A) [36]. Furthermore, IL-10, an immunosuppressive factor, plays a critical role in inhibiting inflammatory responses and promoting tissue repair [82]. Mg^2+^ can increase the secretion of IL-10 by M2 macrophages and inhibit OC-mediated bone resorption through the IL-10 signalling pathway (Figure 7B and C) [35, 36]. Research by Lin *et al.* demonstrated that Mg^2+^ can increase the proportion of Arg1-positive cells (M2 macrophages) and decrease the proportion of iNOS-positive cells (M1 macrophages) at injury sites (Figure 7D and E) [35]. Moreover, Mg^2+^ can reduce the number of M1 macrophages and inflammation by inhibiting the activation of the TLR4 signalling pathway, thereby suppressing OC-mediated bone resorption [83].

Second, Mg^2+^ can influence the metabolic state and energy metabolism of macrophages, thereby affecting their immunological characteristics and functions. Mg^2+^ promotes mitochondrial oxidative phosphorylation in macrophages, leading to increased ATP production and cellular stability. Additionally, Mg^2+^ regulates lipid metabolism and oxidative stress in macrophages, which impacts their capacity for tissue repair and regeneration [84–86].

Finally, studies have shown that Mg^2+^ also inhibits OC-mediated bone resorption. Mg^2+^ can inhibit OC activity and bone resorption through various pathways, such as reducing the secretion of inflammatory cytokines such as TNF-α and IL-1 by macrophages. These inflammatory mediators can activate the NF-κB pathway, stimulating OC secretion of lysosomal enzymes, thereby accelerating bone resorption and contributing to conditions such as osteoporosis [3, 74].

In summary, Mg^2+^ plays a crucial regulatory role in the immune system, particularly in the polarization and function of macrophages [87, 88]. Future research should explore the mechanisms of Mg^2+^ in immune regulation to discover more precise and effective therapeutic strategies, providing new insights into the treatment of osteoporosis and other bone-related diseases.

## Mg affects OCs by regulating hormone and peptide levels

Bone resorption is a complex process that is coordinated by various factors; it is influenced not only by local cytokines, inflammatory factors and mechanical factors but also by systemic hormones and peptides, such as parathyroid hormone (PTH), prostaglandins (PGs) and calcitonin [89–91]. As the second most abundant intracellular cation in the human body, Mg^2+^ participates in hundreds of biochemical reactions and enzyme-catalysed processes, significantly influencing the levels of various hormones in the body; it may regulate OC formation by affecting the release of multiple hormones [92–94].

**Figure 8.**
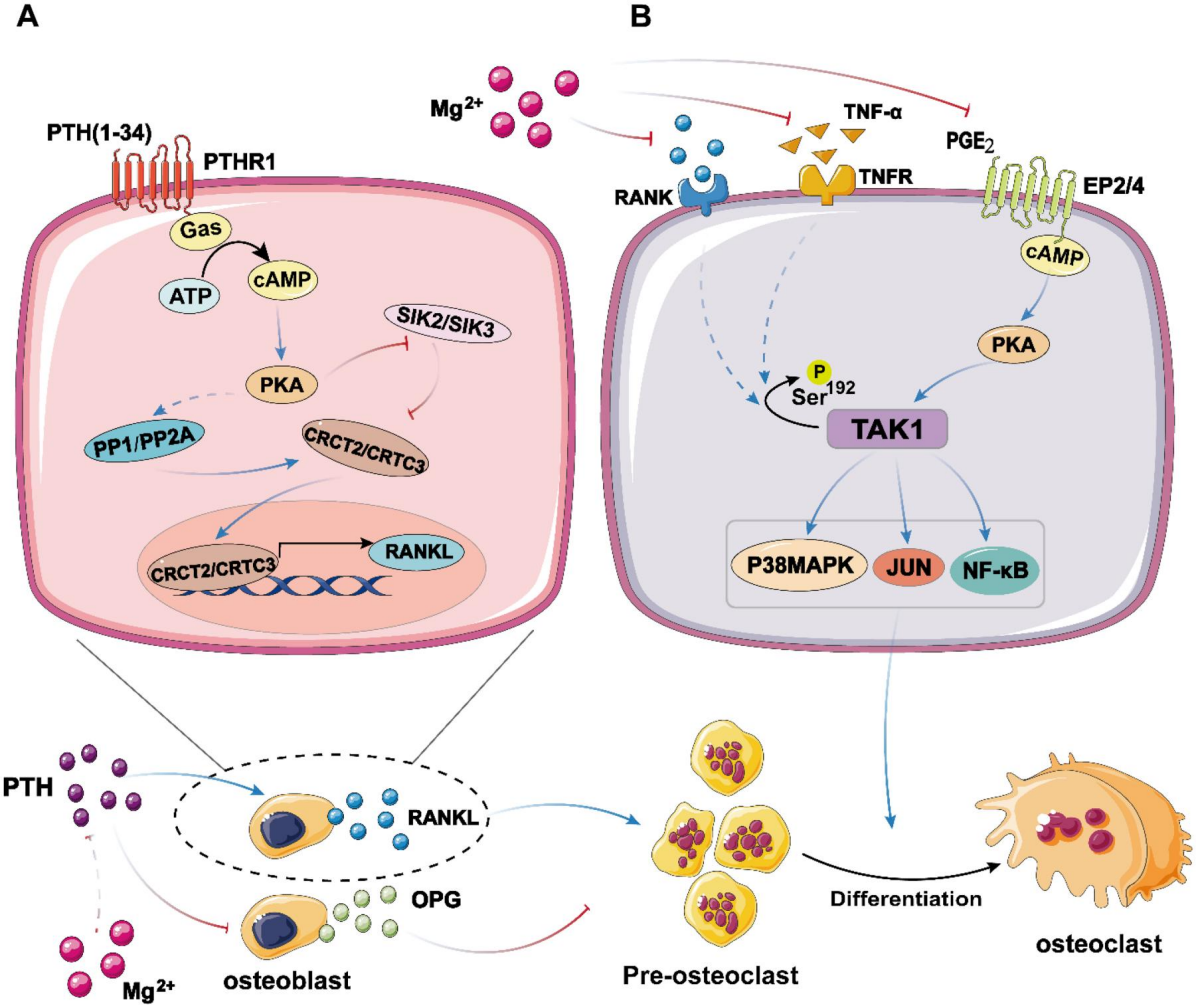
(**A**) PTH reduces the binding of RANKL to OPG by inhibiting the secretion of OPG from osteoblasts and acting on the cAMP-PKA-CREB and PP1/PP2A-CRTC3 pathways. Therefore, it regulates osteoclast generation. Mg^2+^ indirectly promotes the formation of osteoclasts by inhibiting PTH production. (**B**) PGE_2_ promotes osteoclast generation through the cAMP/PKA signalling pathway. Mg^2+^ inhibits osteoclast generation by inhibiting PGE_2_ production.

Mg^2+^ plays an important role in the secretion of PTH. PTH is one of the main hormones regulating the balance of calcium and phosphorus, and an increase in its secretion promotes the formation and activation of OCs and accelerates the bone absorption process. Magnesium ions indirectly regulate the activity of OCs by regulating the secretion of PTH. Studies have shown that intermittent PTH release promotes bone formation, whereas continuous PTH release leads to bone resorption [95–97]. This process may be mediated through the activation of signalling pathways such as the cAMP-PKA-CREB, PI3K/AKT/STAT5 and PP1/PP2A-CRTC3 pathways, which promote RANKL expression and OC differentiation [98–100] (Figure 8A). Additionally, PTH inhibits OB secretion of OPG, reducing the chance for RANKL to bind with OPG, thus amplifying the interaction between RANKL and RANK, which further promotes osteoclastogenesis. Mg^2+^ has been demonstrated to exert a regulatory effect on PTH secretion. When serum levels of free Mg^2+^ decrease (e.g. in postmenopausal women with reduced Mg^2+^ levels in serum and bone), circulating PTH levels may decline [92, 101]. Thus, the elevated serum Mg^2+^ levels due to the degradation of biodegradable magnesium alloy implants might stimulate continuous PTH release, potentially promoting OC formation [93].

PGs act as functional regulators of bone metabolism, stimulating both bone formation and bone resorption. PGs can upregulate the expression of RANKL in OBs and downregulate the expression of OPG, thereby promoting the formation of OCs [102]. Research suggests that PGE_2_ may play a critical synergistic role in enhancing RANKL- and inflammatory cytokine-induced bone resorption in OC precursors via the cAMP/PKA signalling pathway, particularly through the activation of TAK1 [102] **(**Figure 8B). Moreover, some reports indicate that PGs can transiently inhibit the activity of mature OCs, which paradoxically influences the bone resorption process [103, 104]. Therefore, PGE_2_ may exert a biphasic effect on osteoclastogenesis, with an initial inhibitory phase followed by a later stimulatory phase of OC formation [104]. Research has also shown that Mg^2+^ can promote PG synthesis by activating desaturase, an enzyme essential for PG production [94]. Thus, the regulation of PG synthesis by Mg^2+^ may be an important indirect factor affecting OC-mediated bone resorption.

Other related studies have shown that calcitonin gene-related peptide (CGRP) plays a crucial role in inhibiting bone resorption. CGRP can suppress OC-mediated bone resorption by inhibiting OC motility [105, 106]. Additionally, the release of magnesium ions can promote new bone formation by increasing the expression of CGRP [2]. Additionally, amylin and calcitonin have been shown to inhibit OC formation [106, 107]. Therefore, whether magnesium ions potentially regulate these hormones and peptides may also impact OC formation.

Notably, whether the level of magnesium ions released during the degradation of biodegradable magnesium-based materials is sufficient to have potential effects on hormone and peptide levels is still worth our attention. Professor Qinling's team confirmed that the implantation of magnesium-loaded intramedullary nails (Mg-IMNs) into the bone marrow of rats can effectively promote the expression of CGRP and thus improve fracture healing in these rats [2]. However, there is a relative lack of experimental research on the potential impact of biodegradable magnesium implants on hormone and peptide levels, and further studies are needed to demonstrate how released magnesium ions influence OCs through endocrine pathways.

Although there has been preliminary progress in understanding how magnesium regulates hormone levels to influence OCs, many specific mechanisms remain unclear. Future research needs to explore in greater depth how Mg specifically affects OC signalling pathways and how these effects can be translated into clinical applications, such as in the prevention and treatment of osteoporosis. This area is worthy of further investigation.

## Mg-based biomaterials affect OCs by releasing hydrogen

Hydrogen (H_2_) has received much attention because of its antioxidant, anti-inflammatory and antiapoptotic properties. H_2_ is a highly selective antioxidant that effectively cleans harmful oxygen free radicals, thereby protecting cells from oxidative stress [108] (Figure 8A). With increasing research, the application potential of H_2_ in the treatment of osteoporosis, arthritis and other bone diseases has gradually emerged. H_2_ has shown marked effects and prospects, especially in the regulation of OCs. H_2_ is an important degradation product of biodegradable magnesium-based implants, and it has been shown that the H_2_ (1460 ± 320 µM) produced by magnesium-based implant degradation as a fracture fixator is much greater than that produced by magnesium-based implant degradation in subcutaneous tissue (550 ± 210 µM) and the skin surface (120 ± 50 µM) [109]. In addition, the concentration of H_2_ measured in the bone marrow was also greater than that in the saturated aqueous solution of H_2_ [109]. Therefore, whether H_2_ has a potential effect on bone healing and the bone remodelling process deserves attention.

H_2_ can inhibit oxidative stress and reduce oxidative damage in cells, thus protecting the normal function of OCs [110, 111]. ROS have been confirmed to play important regulatory roles in OC differentiation [112–114]. On the one hand, reactive oxygen species (ROS) can induce OB apoptosis, thereby reducing the production of OPG. In this way, the competitiveness of OPG is weakened compared with that of RANKL, and the blocking effect on the combination of RANK and RANKL is reduced, which promotes the differentiation of OCs [115]. On the other hand, ROS can induce the self-amplification of NFATc1 to promote OC fusion and functional gene expression [116]. Oxidative stress is an important factor in OC activation, and H_2_ can effectively remove ROS in OCs and reduce oxidative stress; it can effectively inhibit the production of intracellular ROS through NADH oxidase 1 (Nox1) [117]. Therefore, the antioxidant effect of H_2_ can effectively inhibit the overactivation and bone resorption of OC. Therefore, the inhibition of ROS production may be an important way for H_2_ to further regulate OC generation.

**Figure 9.**
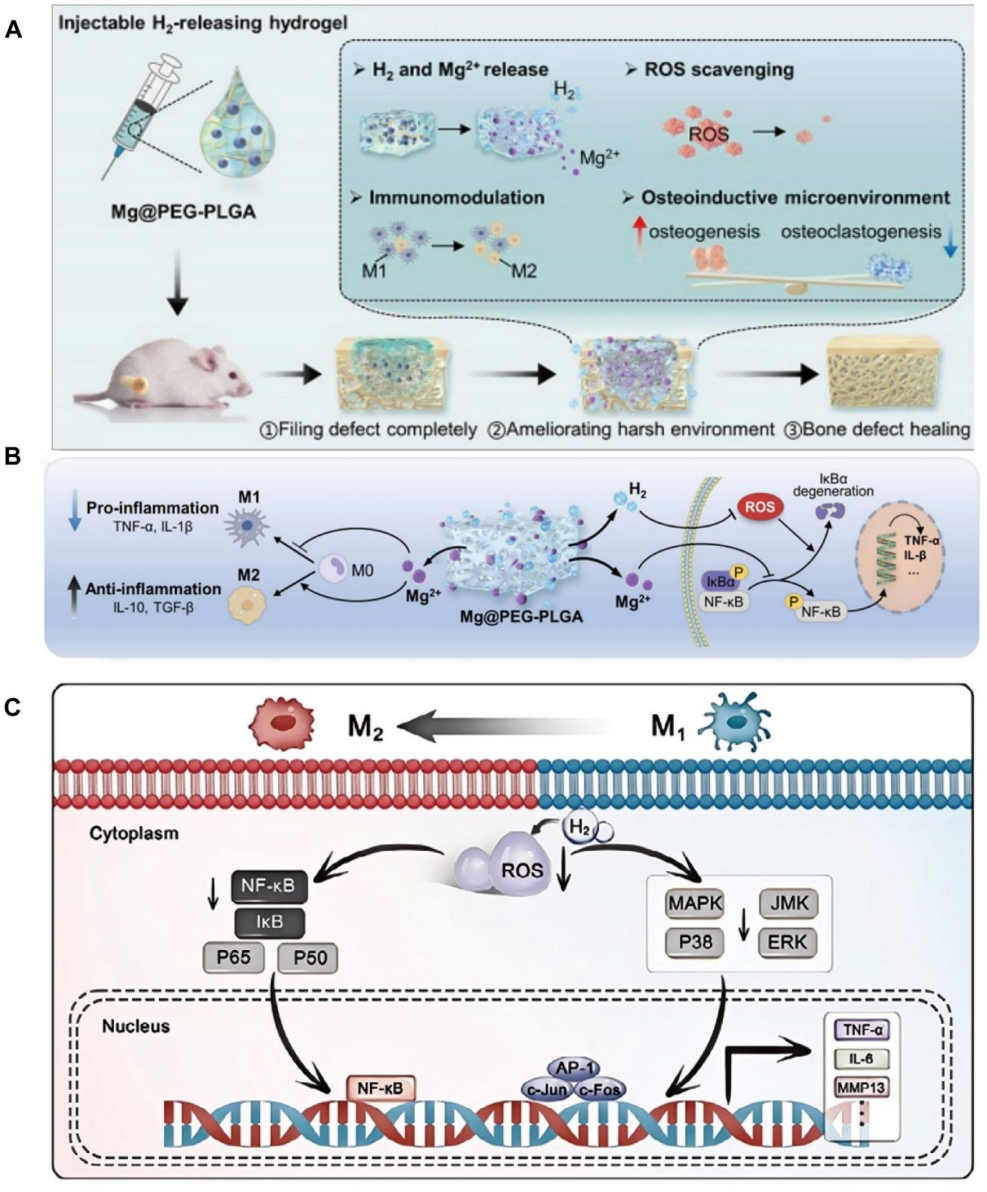
(**A**) Schematic of the mechanism by which degradable Mg@PEG-PLGA hydrogel effectively promotes osteoplastic bone regeneration. Figure is licenced under CC by-NC-ND 4.0. [121]. (**B**) The Mg-@PEg-PLga hydrogel exerts significant anti-inflammatory effects by modulating macrophage polarization and inhibiting the IκBα/NF-κB pathway. Figure is licenced under CC by-NC-ND 4.0 [121]. (**C**) Hydrogen inhibits NF-κB by clearing the ROS BB and MAPK signalling pathways and then downregulates the expression of inflammatory factors (TNF-α, IL-6, MMP-13, etc). Adapted and reproduced from Ref. [120]. Copyright © 2024 Wiley‐VCH GmbH.

H_2_ can affect the differentiation and proliferation of OCs by regulating cell signalling pathways, such as the RANKL/OPG signalling pathway and the PI3K/Akt and Nrf2/ARE pathways, thus affecting the formation and repair process of bone tissue [118, 119]. Li *et al.* [117] used H_2_ to treat RAW264.7 cells and promote OC differentiation. First, H_2_ inhibited the ability of the BMMs to absorb lacunae; second, it reduced the mRNA expression of OC-related specific marker genes (TRAP, Cathk, Calcr, etc) and last, it inhibited the protein expression of NFATc1 and c-Fos, as well as the activity of NF-kB and the activation of MAPK and other pathways. Similarly, Shi *et al.* [120] also found that H_2_ can downregulate the MAPK and NF-κB signalling pathways (Figure 9C). This finding was further confirmed in the experiments conducted by Zhou *et al*. [121] (Figure 9B). Liu *et al.* reported through *in vitro* studies that H_2_ can significantly inhibit the expression of genes related to OC generation (Ctsk, Calcr and Mmp9). In addition, H_2_ can inhibit the proliferation of BMMCs in OC induction medium (including M-CSF and RANKL) in mice and promote apoptosis [122].

H_2_ has a significant effect on the function of macrophages. M1 macrophages play a key role in the inflammatory response. They exacerbate local inflammation by secreting proinflammatory factors and play important roles in the occurrence and development of bone diseases. M2-type macrophages promote bone repair and remodelling by secreting anti-inflammatory factors. H_2_ inhibits the release of TNF-α, IL-6 and other inflammatory factors by M1 macrophages, effectively inhibiting the inflammatory response of M1 macrophages [120] (Figure 9A and C). These effects promote the polarization of M2-type macrophages, increase their ability to secrete anti-inflammatory factors, reduce the level of inflammation and inhibit the activity of OCs; this slows the process of bone destruction and helps control the development of related bone diseases, e.g. rheumatoid arthritis.

In conclusion, H_2_ released during Mg-based implant degradation may have a certain inhibitory effect on OC formation. Therefore, further exploration of the molecular mechanism by which H_2_ affects OC activity and the identification of H_2_ targets and signalling pathways are necessary.

## Challenges and prospects

With excellent biodegradability and biocompatibility, magnesium-based materials have shown great application potential in the field of orthopaedics. In particular, these materials exhibit unique multitarget regulatory effects that canin bone repair and regeneration, effectively promoting bone formation and while inhibiting bone resorption. Magnesium-based materials not only play important roles in fracture repair, bone defects, osteoporosis and other common orthopaedic diseases but also present significant advantages in personalized treatment. By precisely controlling the degradation rate and release behaviour of magnesium ions, combined with advanced technologies such as 3D printing and functional coatings, the potential for personalized applications of magnesium-based materials is further enhanced.

### Magnesium-based materials regulate the immune response and promote bone repair

Research on bone tissue implant materials has gradually changed from immune-friendly materials to immune-regulated materials. Increasing research has elucidated the close interactions between OCs and macrophages. OCs and macrophages are both important components of the bone marrow and play key roles in the regulation of bone metabolism; they regulate the physiological function and metabolic balance of bone tissue through a variety of cytokine interactions and play important roles in the occurrence and development of bone diseases such as osteoporosis, rheumatoid arthritis and bone tumours. The use of bioactive materials to regulate the immune response of macrophages to promote bone healing has become a research hotspot in the field of bone repair and regeneration in recent years. The excessive secretion of inflammatory factors is an important factor leading to the overactivation of OCs. Mg^2+^ released by Mg-based materials can build an immune microenvironment that promotes bone formation by promoting the recruitment and polarization of macrophages. This process not only promote the transformation of macrophages from the M1 phenotype to the anti-inflammatory M2 phenotype but also inhibit the proinflammatory factors TNF-α and IL-1β and upregulate the expression of the anti-inflammatory factor IL-10 and other related inflammatory factors, thereby inhibiting the formation of OCs. H_2_ released during Mg-based material degradation can also inhibit the release of inflammatory factors by M1-type macrophages, promote the polarization of M1-type macrophages to the M2 type [111], promote the release of anti-inflammatory factors by M2 macrophages and effectively slow bone destruction. The degradation products of biodegradable magnesium-based materials can not only promote bone formation but also effectively inhibit bone resorption through the regulation of the immune microenvironment, macrophage polarization and OCs, providing dual advantages for bone repair [87, 123]. This strategy of regulating the immune response with bioactive materials provides novel ideas for bone repair and regeneration.

### Risks to cells and tissues from products of magnesium-based material degradation

In the research and application of magnesium-based materials, the release behaviour of magnesium ions and hydrogen in the degradation process directly affects their biological effects and clinical application effects. Magnesium ions can promote bone repair dose-dependently at appropriate concentrations. The degradation rate of materials affects the concentration of magnesium ions *in vivo*, and an appropriate concentration of magnesium ions helps maintain the physiological function of cells and promotes their activity. For example, HP-Mg and ZK60/MBG materials resulted in a low degradation rate (approximately 0.18 mm/year), and suitable magnesium ion concentrations around the materials promoted MG-63 cells and mineralization. Similarly, magnesium alloys (such as ZQ71) release magnesium ions through slow degradation, resulting in good cell proliferation and promoting new bone formation (Table 3). High concentrations may lead to cytotoxicity, inflammation and even nerve damage. Although hydrogen has antioxidant and anti-inflammatory effects, an excessive release rate may lead to the formation of air sacs, affect the contact between implants and surrounding tissues, delay bone healing and even cause mechanical compression and tissue damage. Therefore, accurately controlling the release rate of magnesium ions and hydrogen to ensure their safety and effectiveness in biomedical applications has become a core topic of current research.

**Table 3.** Degradation performance and biocompatibility of magnesium-based materials *in vitro* and *in vivo*

Materials	*In vitro*		*In vivo*			Refs.
	Degradation rate	Effects on cells	Implantation duration	Rate of corrosion or degradation	New bone formation (yes/no)	
Pure Mg	A week: 0.75 ± 0.45 mm/year Four weeks: 0.32 ± 0.04 mm/year	Good primary and pre osteoblast cell viability	84 days	Less than 0.2 mm/year	Yes	[124]
HP-Mg	0.18 mm/year					[125]
UHP-Mg	0.22 mm/year					[125]
As-cast Mg–2Zn			84 days	Approximately 0.3 mm/year		[126]
R3–91% Mg–2Zn	1.0 mg/cm^3^/d		84 days	0.2 mm/year		[126]
HPMg			84 days	About 0.18 mm/year		[126]
ZK60/BG	0.52 ± 0.11 mm/year	MG-63 activity increased				[127]
ZK60/MBG	0.31 ± 0.13 mm/year	MG-63 activity increased				[127]
Mg-PLLA-G	0.51 ± 0.06 mm/year	MG-63 activity increased				[128]
WEJ431	11.1 ± 0.4 mm/year	Promoted BMSC proliferation	60 days	1.04 ± 0.31 mm/year	Yes	[129]
FSP800-WEJ431	0.4 ± 0.1 mm/year	The promotion effect on BMSC proliferation was more obvious	60 days	0.09 ± 0.03 mm/year	Yes	[129]
MgF_2_ coated AZ31	6.65 × 10^−7^ A cm^−2^	The effect on BMSC adhesion was more obvious				[130]
Bare AZ31	2.81 × 10^−6^ A cm^−2^	The survival rate of MC3T3 cells was greater than 75%				[130]
JDBM	0.337 mm/year	Promoted the proliferation of EA.hy 926 cells				[131]
MgF_2_-coating JDBM	0.253 mm/year	The promotion effect on the proliferation of EA.hy 926 cells was more obvious				[131]
EBPed ZKX50	2.36 mg/cm^3^/days					[132]
EBPed-HT ZKX50	0.46 mg/cm^3^/days					[132]
ZQ71	17.1 ± 6.1 mg/cm^3^/days	Promoted the proliferation of MC 3T3-E1 cells	32 days	46.6 ± 3.1 mg/cm^3^/days	Yes	[133]
ZQ63	17.0 ± 4.0 mg/cm^3^/days	Promote the proliferation of MC 3T3-E1 cells	39 days	54.1 ± 2.8 mg/cm^3^/days	Yes	[133]
Bare AZ31	2.81 × 10^−6^ A cm ^−2^	The survival rate of MC3T3 cells was greater than 75%				[134]
Na-MMT coating AZ31	2.90 × 10^−7^ A cm ^−2^	The survival rate of MC3T3 cells was greater than 80%				[134]
Zn-MMT coating AZ31	2.86 × 10^−7^ A cm ^−2^	The survival rate of MC3T3 cells was greater than 80%				[134]
Mg–2.0 Zn–1.0 Gd	0.24 mm/year	Cytotoxicity to MC3T3-E1 cells was 0–1	30 days	0.31 mm/year	Yes	[135]

First, controlling the release rate and total amount of magnesium ions during the degradation of magnesium-based materials is a key technical challenge. An excessive degradation rate may lead to excessive local magnesium ion concentrations around the material, which may cause cytotoxicity or adverse inflammatory responses. The biological effects of magnesium differ from those of several heavy metal ions (such as mercury and lead), the toxicity of which is closely related to their accumulation in the body and ability to inhibit important enzymes and proteins. Severe neurocytotoxicity can be induced even at low concentrations, and their toxicity is irreversible. However, the toxicity of Mg^2+^ is mainly dose dependent, and only when the concentration of Mg^2+^ exceeds a certain threshold can it cause adverse effects on cells, tissues and nerves [136] (Table 4). Our previous studies revealed that when the concentration of Mg^2+^ is 5–10 mM, it can significantly promote cell proliferation, and when the concentration exceeds 20 mM, it has an inhibitory effect [1]. This finding is also consistent with most experimental studies. Low to moderate concentrations (0.8–10 mM) of magnesium ions significantly promote mineralization and the expression of osteogenic genes (such as ALP, RUNX2 and OCN); in particular, hFOB1.19 cells, BMSCs and MG63 cells exhibit the greatest effects at concentrations ranging from 5 to 10 mM (Table 4). However, the concentration range of magnesium ions on OCs is relatively unknown at present. Liu *et al.* reported that when the concentration of magnesium ions is less than 3 mM, the inhibitory effect on RAW264.7 cells is extremely weak, and when the concentration is greater than 6 mM, the activity of RAW264.7 cells is reduced. However, mature OCs can tolerate high concentrations of magnesium ions (16 mM), with little effect on cell numbers. Similarly, Yeung *et al.* [3] reported that highly soluble magnesium ions (8 mM) promote OC formation by significantly downregulating gene expression. However, Anne *et al.* [143] did not find a significant promotion effect on human primary OBs, OBs or OCs after triple culture in the presence of extracts containing 5 and 10 mM magnesium. In addition, magnesium plays a duel role in the nervous system: at appropriate concentrations, magnesium helps maintain the homeostasis of neurons and can play a certain neuroprotective role after injury [144]; however, excessive magnesium ion concentrations may have toxic effects on neurons, resulting in impaired nerve function, possibly triggering metabolic disorders in nerve cells and even accelerating cell death. This dose-dependent feature provides a certain degree of flexibility for the safety of magnesium-based materials. Accurately controlling the release rate of magnesium ions can make full use of its good bone-promoting and bone-breaking characteristics without causing toxicity. Therefore, accurately ascertaining the optimal Mg^2+^ concentration is worthy of in-depth exploration. Although *in vivo* studies of magnesium-based materials have shown that Mg^2+^ has a significant effect on OBs and OCs, there is still a lack of in-depth systematic studies. Many studies have focused on the degradation behaviour of magnesium-based materials *in vitro* and the effects of Mg^2+^ release on bone metabolism. However, *in vivo* experiments involve complex biological environments and multiple interactions, and the precise control of the magnesium ion release rate, local concentration in implants, and long-term tracking of cell behaviour remain challenging.

**Table 4.** Effects of different Mg^2+^ concentrations on cell differentiation

Mg^2+^ concentration	Cell type	Effects on cells and gene expression		Refs.
0.8, 2, 5, 10 and 20 mM 40 mM	hFOB1.19 cells	Osteoblast mineralization and ALP expression	5, 10, 20 mM: significantly (+); 40 mM (−)	[1]
0.8 and 8 mM	BMMs	TNF-α and IL-1β	8 mM (−)	[3]
0.8 and 8 mM	THP1-derived macrophages	Trap, RANK and M-CSF	8 mM (+)	[3]
0.8 and 8 mM	MSCs	ALP, COL1A1, OPN, IBSP, BGLAP and Osterix	8 mM (+)	[3]
1.6, 2.5, 5, 10 and 20 mM	rBMSCs	ALP and OCN	<10 mM (+); 2.5 and 5 mM: most significantly (+)	[137]
0.8, 5, 10 and 20 mM	hBMSCs	Extracellular mineralization	5 and 10 mM: most significantly (+)	[138]
0.8, 1.8, 5, 10 and 20 mM	Periosteum-derived cells	Extracellular mineralization; RUNX2, BSP and OCN	5, 10 and 20 mM: significantly (+)	[139]
1, 3 and 6 mM	MG63 cells	ALP, RUNX2 and OCN	3 mM: significantly (+) 6 mM: most significantly (+)	[140]
10 mM	MC3T3-E1 cells	Extracellular mineralization; ALP expression	10 mM: significantly (+)	[141]
5 and 10 mM	BMSCs	ALP expression	5–10 mM significantly (+)	[142]

Second, when magnesium-based materials degrade too fast, a certain amount of hydrogen is produced locally. Hydrogen itself is considered bioinert in the body, without known toxicity to cells or tissues; additionally, it is beneficial to cells and tissues through its antioxidant, anti-inflammatory and cell repair effects [145]. However, when the hydrogen production rate is greater than the absorption rate of the human body, hydrogen will not be absorbed in a timely manner and will be dispersed, which will lead to hydrogen accumulation around implants. The formation of local air sacs not only affects the close contact of an implant with surrounding tissue but also may lead to limited local blood circulation, further affecting the supply of oxygen and nutrients and thus delaying the bone healing process. In addition, excessive gas accumulation can lead to mechanical compression, damage the surrounding soft tissue and bone structure, increase the risk of infection, and, in extreme cases, trigger an inflammatory response and tissue necrosis. Therefore, controlling the rate of hydrogen production is a key factor in ensuring the safety and effectiveness of magnesium-based materials in biomedical applications.

In addition, inconsistent degradation may affect the structural integrity and mechanical properties of magnesium-based materials. *In vivo*, the degradation of magnesium-based materials is usually affected by the local acid–base environment, stress distribution and the composition of the material itself, resulting in a non-uniform degradation. This non-uniform degradation can cause the local corrosion of material, thus affecting the stability, load-bearing capacity and thus the long-term use of the implant. Controlling the degradation rate of magnesium-based materials to ensure an ideal mechanical properties throughout the implantation period is also an important challenge in the research and application of magnesium-based materials. Therefore, fine regulation of the degradation rate is the key to develop safe and effective magnesium-based biomaterials.

### Mg-based material degradation regulation, biosafety optimization and risk prevention strategies

Using material design and regulating release kinetics to solve the excessive local magnesium ion concentration caused by the rapid degradation of magnesium-based materials, excessive gas production around the implant and non-uniform degradation have become key topics of current research.

Determining the concentration control window of magnesium ions is important. Magnesium ions can promote the activity of OBs [146], and a concentration that is too high may completely inhibit the activity of OCs, resulting in insufficient bone absorption and affecting bone remodelling; thus, Mg^2+^ is particularly important for the regulation of OC activity. The key is to find a magnesium concentration window that can promote osteogenesis while retaining certain OC activities to achieve a bone remodelling balance. According to existing studies, a concentration range between 3 and 10 mM may be a key window for balancing osteogenic and osteoclastic activities; however, this needs to be verified by *in vitro* and *in vivo* experiments. For example, OBs (MC3T3-E1) were cocultured with OCs (RAW264.7) to evaluate ALP activity, bone nodule formation and the number of TRAP-positive cells at different magnesium concentrations. Anne *et al.* [143] conducted *in vitro* triple cultures of human osteoprogenitor cells, OBs and OCs in the presence of extracts containing 5–10 mM magnesium. Histological analysis (HE staining, TRAP staining) was performed to evaluate the dynamic balance of bone formation and absorption.

Monitoring the degradation rate of Mg-based materials and the concentration of magnesium ions is a major challenge, particularly *in vivo* applications where local concentration monitoring post-implantation is critical. The complexity of *in vivo* monitoring necessitates the acquisition of accurate, real-time data, as opposed to relying solely on simplified *in vitro* simulation results. It is necessary to fully consider the complexity of the internal environment, such as the influence of fluid flow, tissue wrapping and other factors on monitoring. To accurately monitor the local magnesium ion concentration released by magnesium-based implants after implantation *in vivo*, a variety of real-time, dynamic detection techniques can be combined. For example, microdialysis technology—utilizing microdialysis probes near the implantation site and the continuous collection of local tissue fluid combined with inductively coupled plasma–mass spectrometry (ICP-MS) or atomic absorption spectrometry (AAS) through a semipermeable membrane. This approach allows for direct assessment of magnesium ion concentration in the microenvironment around an implant. Implantable electrochemical microsensors based on magnesium ion-selective membranes can monitor local magnesium ion concentration changes in real time (sensitivity up to μM). In addition, microcomputed tomography (μCT) can be used to observe dynamic changes in implant morphology (such as porosity and volume loss), and the degradation rate can be quantified by three-dimensional reconstruction [147–149]. In the future, machine learning algorithms can be used to analyse sensor data to identify patterns of magnesium concentration changes at different stages of degradation; it can also be combined with image data generated during the degradation of magnesium-based materials (such as MRI, CT or fluorescence imaging) and computer vision technology. Imaging data can be analysed through deep learning to track the changes in the local magnesium ion concentration during degradation and its influence on surrounding tissues. This approach can greatly improve the accuracy of monitoring.

By monitoring the *in vivo* concentration of magnesium ions, we can gain valuable insights into the effects of ion release from magnesium-based materials at various degradation stages on bone remodelling. This approach helps mitigate potential risks caused by elevated magnesium ion levels and supports the development of materials with enhanced biosafety.

### Design strategy for Mg-based medical materials on the basis of their characteristics and properties

The design of magnesium-based medical implant materials should be closely combined with the biodegradation characteristics of the materials and the performance requirements of orthopaedic clinical applications. To improve the application effect of magnesium-based implants, optimizing the degradation rate and precisely regulating the release behaviour of magnesium ions are crucial.

Alloying design is one of the core strategies of magnesium-based materials [150]. By introducing alloying elements such as zinc (Zn) and strontium (Sr), the electrochemical activity of the magnesium matrix can be effectively influenced. Zn, Sr and other elements can not only reduce the degradation rate but also promote the fusion and maturation of OC precursors, inhibit OC differentiation and maintain bone homeostasis [151–153]. Through this alloy design, it is possible to reduce the accumulation of excessive degradation products, such as hydrogen, to avoid negative effects on surrounding tissues while ensuring the biocompatibility of magnesium ion release, effectively supporting the bone repair process.

In terms of surface functional coating design, magnesium-based materials can be used for targeted therapy by loading them with slow-release drugs (such as bisphosphonates, siRNAs, etc), regulating the activity of OCs and directly interfering with bone metabolism [154]. Responsive coatings (such as pH-sensitive, enzyme-sensitive or pH-enzyme-sensitive coatings) can trigger coating degradation in low pH environments where local inflammation or OCs are active, releasing Mg^2+^ as needed to avoid toxicity to surrounding tissues due to high Mg^2+^ concentrations [155–157]. Moreover, the coating can be designed to avoid the accumulation of a large amount of hydrogen in a short period of time, reducing its adverse effects on the tissue, such as pressure and gas swelling, thereby improving the safety of the implant. In addition, the combination of electrospinning technology with nanocomposite coating technology also has significant advantages. For example, Mostaan *et al.* [128] deposited PLLA/GO-AgNPs (poly-L-lactic acid/graphene oxide-silver nanoparticles) with different concentrations of GO-AgNPs on a Mg alloy to effectively slow the degradation rate of the Mg alloy matrix, reduce the H_2_ release rate, reduce the pressure on surrounding tissues and simultaneously have good antibacterial properties.

The gradient design of composite materials is an effective means to further improve the properties of Mg-based materials [158–160]. Core–shell design is a common gradient material design strategy, with a magnesium-based material as the core and a controlled degradation polymer (such as polycaprolactone PCL) or ceramic (such as β-tricalcium phosphate (β-TCP)) as the outer layer [161]. In the early stage of implantation, the outer layer degrades first, releasing a small amount of Mg^2+^and hydrogen, inhibiting the early inflammatory reaction and reducing the activity of OCs. The inner layer slowly degrades, and the long-term stable release of Mg^2+^ and hydrogen prevents the accumulation of hydrogen in the surrounding tissues. Through this gradient design, the release rate of magnesium ions and hydrogen can be precisely controlled, improving the stability and long-term efficacy of implants. In addition, 3D printing technology can be used to design a gradient pore structure that meets the permeability characteristics of biological fluids and gas diffusion requirements, further optimizing the uniform release of magnesium ions and the control of hydrogen, thus promoting the bone repair process.

In other innovations in material design, an ion exchange carrier system has been used to load magnesium ions in bioactive glass or layered double hydroxide (LDH) through an ion exchange mechanism to achieve the slow release of Mg^2+^, further maintaining the ideal concentration window for osteogenesis and the inhibition of OCs [162, 163]. It is also possible to design microspheres/nanoparticles to encapsulate Mg-based materials in polylactic acid–glycolic acid (PLGA) microspheres and regulate the release of Mg^2+^ through the degradation rate of the microspheres [164, 165]. In addition, multiphase structure designs, particle enhancement and distribution optimization design also show great potential. For example, Swain *et al.* [166] prepared Ca_8_Mg_2_(PO_4_)_6_(OH)_2_-gelatine (MgHA-GEL) bone-filling material via the freeze–drying method, and the material exhibited good osteogenic properties. These technical solutions provide great development potential for the application of magnesium-based materials in the field of orthopaedics and can be used to precisely regulate the release of magnesium ions to meet the needs of different orthopaedic surgeries.

Finally, to overcome the degradation of the mechanical properties of Mg-based materials during degradation, the mechanical strength of Mg-based materials can be enhanced by refining the grain and designing micropore structures [167, 168]. In the process of degradation, a refined grain can effectively increase the strength of a material, and a reasonable pore structure design can promote the uniform release of gas to avoid structural instability caused by excessive local degradation. Through material alloying, surface coating, composite gradient design, release dynamics regulation and other aspects of optimization, the precise regulation of magnesium-based implant materials can be achieved; these materials will not only inhibit OC activity but also promote osteogenesis and ultimately improve the long-term stability and bone integration efficiency of implants, meeting the high requirements for materials in orthopaedic clinical applications.

### Precise release and personalized application: the development direction of biodegradable magnesium-based materials in the treatment of orthopaedic diseases

Biomaterials containing magnesium (including magnesium alloys, bioceramics containing magnesium, hydrogels containing magnesium, magnesium matrix composites, etc) show a diverse development trend [164, 169–171]. Owing to their degradability and excellent biocompatibility, biodegradable magnesium-based materials have shown extensive application potential in the field of orthopaedics. The released magnesium ions and other products play a multidimensional role in fracture repair, bone defect repair, osteomyelitis, osteoarthritis, osteoporosis and other diseases by regulating the bone metabolic microenvironment (Table 5). In particular, biodegradable magnesium-based materials have shown significant advantages in the personalized treatment of diseases. For example, 3D-printed multifunctional bionic bone scaffolds combined with TP–Mg nanoparticles have been used for the repair of infectious bone defects. A nanosystem that combines ascorbic acid (AA) and MgFe LDH has been shown to be beneficial not only for reshaping the bone immune microenvironment but also for combatting bacterial infection and reducing the inflammatory response, especially in the treatment of osteomyelitis [162]. Similarly, Yang *et al.* [172] used the eddy heat effect of magnesium implants under an alternating magnetic field (AMF) to control the simultaneous release of magnesium ions, hydroxide ions and hydrogen to effectively remove ROS, which inhibited OCs to reduce bone damage, reversed the inflammatory environment and inhibited bacterial proliferation, providing a new perspective and idea for solving deep tissue infection. The Mg@PEG-PLGA gel developed by Zhou *et al.* [121] can reduce intracellular ROS through the precise release of magnesium ions and hydrogen, induce the polarization of macrophages towards the M2 phenotype and inhibit the IκB/NF-κB signalling pathway; moreover, it also significantly promotes the repair of osteoporotic fractures *in vivo*, providing key insights for the precise targeted inhibition of OCs in the treatment of osteoporosis in the future. Another example is a novel magnesium oxide nanocomposite hydrogel developed by Qin *et al.* [179] that successfully solved the challenge of the difficult healing of bone defects caused by drug-related necrosis of the jaw (MRONJ).

**Table 5.** Advantages and application scenarios of various magnesium-containing materials

Material type	Advantage	Application scenario	Refs.
Magnesium alloy	High mechanical strength and controlled degradation	Internal fixation of fractures and repair of large bone defects	[8, 12]
Magnesium matrix composites	Degradable, controllable mechanical properties and anti-inflammatory properties	Bone defect filling, joint prosthesis and osteomyelitis	[172, 173]
Porous magnesium scaffold	Promotes vascularization and bone integration	Critical size bone defect repair	[174, 175]
Magnesium-based bioceramics	High bone conductivity and antibacterial properties	Osteomyelitis, osteoporosis, etc	[121, 162, 176, 177]
Magnesium based hydrogel	Injectable, anti-inflammatory properties and bone repair	Osteoarthritis and cartilage repair	[158, 177–179]

Biodegradable magnesium and its products have unique advantages in the treatment of orthopaedic diseases through multitarget regulation (osteogenic promotion, osteoclastic inhibition, immune regulation and antibacterial activity) [180]. Moreover, combined with new technologies such as 3D printing and functional coating, the potential of personalized treatment with biodegradable magnesium has become a reality. The clinical application of high-purity magnesium screws in China and MAGNEZIX screws in Germany also highlights the safety and effectiveness of such materials. In the future, it is necessary to further optimize the dynamic balance between the magnesium-based material degradation rate and bone regeneration and further explore the mechanism of action in the complex bone immune microenvironment to promote personalized and precise treatment with biodegradable magnesium in the field of orthopaedics.

## Conclusion

This review addresses the research progress on biodegradable Mg-based materials as orthopaedic implants and summarizes the regulatory mechanism of OCs in biodegradable Mg-based implants. Specifically, biodegradable Mg-based materials play important roles in OC fusion, adhesion, the OPG/RANK/RANKL signalling pathway and immune regulation. Biodegradable magnesium not only promotes bone regeneration by promoting the activity of OBs but also inhibits excessive bone resorption through the regulation of OCs; therefore, it can achieve a balance in bone metabolism, promote bone repair and prevent the excessive resorption of bone defects. Finally, the risk of degradation products to cells and tissues, the regulation of degradation and biosafety optimization of magnesium-based materials, design strategies and development trends of precise magnesium release and personalized application are discussed. In general, research on biodegradable magnesium as an orthopaedic implant material has made remarkable progress, laying a solid foundation for the development of safer and more effective orthopaedic implant materials in the future, thus leading to better treatment results and quality of life for patients.

## References

[1] Zhang X , Zu H, Zhao D, Yang K, Tian S, Yu X, Lu F, Liu B, Yu X, Wang B, Wang W, Huang S, Wang Y, Wang Z, Zhang Z. Ion channel functional protein kinase TRPM7 regulates Mg ions to promote the osteoinduction of human osteoblast via PI3K pathway: in vitro simulation of the bone-repairing effect of Mg-based alloy implant. Acta Biomater 2017;63:369–82. 28882757 10.1016/j.actbio.2017.08.051

[2] Zhang Y , Xu J, Ruan YC, Yu MK, O'Laughlin M, Wise H, Chen D, Tian L, Shi D, Wang J, Chen S, Feng JQ, Chow DH, Xie X, Zheng L, Huang L, Huang S, Leung K, Lu N, Zhao L, Li H, Zhao D, Guo X, Chan K, Witte F, Chan HC, Zheng Y, Qin L. Implant-derived magnesium induces local neuronal production of CGRP to improve bone-fracture healing in rats. Nat Med 2016;22:1160–9. 27571347 10.1038/nm.4162 PMC5293535

[3] Qiao W , Wong KHM, Shen J, Wang W, Wu J, Li J, Lin Z, Chen Z, Matinlinna JP, Zheng Y, Wu S, Liu X, Lai KP, Chen Z, Lam YW, Cheung KMC, Yeung KWK. TRPM7 kinase-mediated immunomodulation in macrophage plays a central role in magnesium ion-induced bone regeneration. Nat Commun 2021;12:2885. 34001887 10.1038/s41467-021-23005-2 PMC8128914

[4] Plaass C , von Falck C, Ettinger S, Sonnow L, Calderone F, Weizbauer A, Reifenrath J, Claassen L, Waizy H, Daniilidis K, Stukenborg-Colsman C, Windhagen H. Bioabsorbable magnesium versus standard titanium compression screws for fixation of distal metatarsal osteotomies – 3 year results of a randomized clinical trial. J Orthop Sci 2018;23:321–7. 29174422 10.1016/j.jos.2017.11.005

[5] Lee JW , Han HS, Han KJ, Park J, Jeon H, Ok MR, Seok HK, Ahn JP, Lee KE, Lee DH, Yang SJ, Cho SY, Cha PR, Kwon H, Nam TH, Han JH, Rho HJ, Lee KS, Kim YC, Mantovani D. Long-term clinical study and multiscale analysis of in vivo biodegradation mechanism of Mg alloy. Proc Natl Acad Sci U S A 2016;113:716–21. 26729859 10.1073/pnas.1518238113 PMC4725539

[6] Zhao D , Huang S, Lu F, Wang B, Yang L, Qin L, Yang K, Li Y, Li W, Wang W, Tian S, Zhang X, Gao W, Wang Z, Zhang Y, Xie X, Wang J, Li J. Vascularized bone grafting fixed by biodegradable magnesium screw for treating osteonecrosis of the femoral head. Biomaterials 2016;81:84–92. 26724456 10.1016/j.biomaterials.2015.11.038

[7] Yu X , Zhao D, Huang S, Wang B, Zhang X, Wang W, Wei X. Biodegradable magnesium screws and vascularized iliac grafting for displaced femoral neck fracture in young adults. BMC Musculoskelet Disord 2015;16:329. 26527162 10.1186/s12891-015-0790-0 PMC4631087

[8] Plaass C , Ettinger S, Sonnow L, Koenneker S, Noll Y, Weizbauer A, Reifenrath J, Claassen L, Daniilidis K, Stukenborg-Colsman C, Windhagen H. Early results using a biodegradable magnesium screw for modified chevron osteotomies. J Orthop Res 2016;34:2207–14. 28005292 10.1002/jor.23241

[9] Kose O , Turan A, Unal M, Acar B, Guler F. Fixation of medial malleolar fractures with magnesium bioabsorbable headless compression screws: short-term clinical and radiological outcomes in eleven patients. Arch Orthop Trauma Surg 2018;138:1069–75. 29696362 10.1007/s00402-018-2941-x

[10] Uzun M. Comparison of magnesium versus titanium screw fixation for biplane chevron medial malleolar osteotomy in the treatment of osteochondral lesions of the talus. Eur J Orthop Surg Traumatol 2020;30:1125. 32253596 10.1007/s00590-020-02657-8

[11] Xie K , Wang L, Guo Y, Zhao S, Yang Y, Dong D, Ding W, Dai K, Gong W, Yuan G, Hao Y. Effectiveness and safety of biodegradable Mg–Nd–Zn–Zr alloy screws for the treatment of medial malleolar fractures. J Orthop Translat 2021;27:96–100. 33520654 10.1016/j.jot.2020.11.007 PMC7807209

[12] Ashammakhi N. CORR Insights^®^: Mg–Zn–Ca alloy (ZX00) screws are resorbed at a mean of 2.5 years after medial malleolar fracture fixation: follow-up of a first-in-humans application and insights from a sheep model. Clin Orthop Relat Res 2024;482:198–200. 37768868 10.1097/CORR.0000000000002866 PMC10723840

[13] Herber V , Labmayr V, Sommer NG, Marek R, Wittig U, Leithner A, Seibert F, Holweg P. Can hardware removal be avoided using bioresorbable Mg–Zn–Ca screws after medial malleolar fracture fixation? Mid-term results of a first-in-human study. Injury 2022;53:1283–8. 34758916 10.1016/j.injury.2021.10.025

[14] Holweg P , Herber V, Ornig M, Hohenberger G, Donohue N, Puchwein P, Leithner A, Seibert F. A lean bioabsorbable magnesium–zinc–calcium alloy ZX00 used for operative treatment of medial malleolus fractures: early clinical results of a prospective non-randomized first in man study. Bone Joint Res 2020;9:477–83. 32874554 10.1302/2046-3758.98.BJR-2020-0017.R2 PMC7437522

[15] Kim JM , Lin C, Stavre Z, Greenblatt MB, Shim JH. Osteoblast–osteoclast communication and bone homeostasis. Cells 2020;9:2073. 32927921 10.3390/cells9092073 PMC7564526

[16] Zhang Z , Zhang X, Zhao D, Liu B, Wang B, Yu W, Li J, Yu X, Cao F, Zheng G, Zhang Y, Liu Y. TGF-β1 promotes the osteoinduction of human osteoblasts via the PI3K/AKT/mTOR/S6K1 signalling pathway. Mol Med Rep 2019;19:3505–18. 30896852 10.3892/mmr.2019.10051 PMC6471541

[17] Seriolo B , Paolino S, Sulli A, Cutolo M. [Are there any positive effects of TNF-alpha blockers on bone metabolism?]. Reumatismo 2006;58:199–205. 17013436 10.4081/reumatismo.2006.199

[18] Abrahamsen B , Shalhoub V, Larson EK, Eriksen EF, Beck-Nielsen H, Marks SC Jr. Cytokine RNA levels in transiliac bone biopsies from healthy early postmenopausal women. Bone 2000;26:137–45. 10678408 10.1016/s8756-3282(99)00260-4

[19] Wang T , He C. TNF-α and IL-6: the link between immune and bone system. Curr Drug Targets 2020;21:213–27. 31433756 10.2174/1389450120666190821161259

[20] Boyce BF. Advances in the regulation of osteoclasts and osteoclast functions. J Dent Res 2013;92:860–7. 23906603 10.1177/0022034513500306 PMC3775372

[21] Liu S , Yan X, Guo J, An H, Li X, Yang L, Yu X, Li S. Periodontal ligament-associated protein-1 knockout mice regulate the differentiation of osteoclasts and osteoblasts through TGF-β1/Smad signaling pathway. J Cell Physiol 2024;239:e31062. 37357387 10.1002/jcp.31062

[22] Udagawa N , Koide M, Nakamura M, Nakamichi Y, Yamashita T, Uehara S, Kobayashi Y, Furuya Y, Yasuda H, Fukuda C, Tsuda E. Osteoclast differentiation by RANKL and OPG signaling pathways. J Bone Miner Metab 2021;39:19–26. 33079279 10.1007/s00774-020-01162-6

[23] Ibáñez L , Nácher-Juan J, Terencio MC, Ferrándiz ML, Alcaraz MJ. Osteostatin inhibits M-CSF+RANKL-induced human osteoclast differentiation by modulating NFATc1. Int J Mol Sci 2022;23:10.3390/ijms23158551 PMC9369336 35955685

[24] Da W , Tao L, Zhu Y. The role of osteoclast energy metabolism in the occurrence and development of osteoporosis. Front Endocrinol (Lausanne) 2021;12:675385. 34054735 10.3389/fendo.2021.675385 PMC8150001

[25] Xue C , Luo H, Wang L, Deng Q, Kui W, Da W, Chen L, Liu S, Xue Y, Yang J, Li L, Du W, Shi Q, Li X. Aconine attenuates osteoclast-mediated bone resorption and ferroptosis to improve osteoporosis via inhibiting NF-κB signaling. Front Endocrinol (Lausanne) 2023;14:1234563. 38034017 10.3389/fendo.2023.1234563 PMC10682992

[26] Jang JS , Hong SJ, Mo S, Kim MK, Kim YG, Lee Y, Kim HH. PINK1 restrains periodontitis-induced bone loss by preventing osteoclast mitophagy impairment. Redox Biol 2024;69:103023. 38181706 10.1016/j.redox.2023.103023 PMC10789640

[27] Rabjohns EM , Hurst K, Ghosh A, Cuellar MC, Rampersad RR, Tarrant TK. Paget's disease of bone: osteoimmunology and osteoclast pathology. Curr Allergy Asthma Rep 2021;21:23. 33768371 10.1007/s11882-021-01001-2

[28] Yang Y , Liu L, Luo H, Zhang D, Lei S, Zhou K. Dual-purpose magnesium-incorporated titanium nanotubes for combating bacterial infection and ameliorating osteolysis to realize better osseointegration. ACS Biomater Sci Eng 2019;5:5368–83. 33464078 10.1021/acsbiomaterials.9b00938

[29] Zheng L , Zhao S, Li Y, Xu J, Yan W, Guo B, Xu J, Jiang L, Zhang Y, Wei H, Jiang Q. Engineered MgO nanoparticles for cartilage-bone synergistic therapy. Sci Adv 2024;10:eadk6084. 38457498 10.1126/sciadv.adk6084 PMC10923500

[30] Li X , Dai B, Guo J, Zhu Y, Xu J, Xu S, Yao Z, Chang L, Li Y, He X, Chow DHK, Zhang S, Yao H, Tong W, Ngai T, Qin L. Biosynthesized bandages carrying magnesium oxide nanoparticles induce cortical bone formation by modulating endogenous periosteal cells. ACS Nano 2022;16:18071–89. 36108267 10.1021/acsnano.2c04747

[31] Zhao Z , Li G, Ruan H, Chen K, Cai Z, Lu G, Li R, Deng L, Cai M, Cui W. Capturing magnesium ions via microfluidic hydrogel microspheres for promoting cancellous bone regeneration. ACS Nano 2021;15:13041–54. 34342981 10.1021/acsnano.1c02147

[32] Li W , Qiao W, Liu X, Bian D, Shen D, Zheng Y, Wu J, Kwan KYH, Wong TM, Cheung KMC, Yeung KWK. Biomimicking bone-implant interface facilitates the bioadaption of a new degradable magnesium alloy to the bone tissue microenvironment. Adv Sci (Weinh) 2021;8:e2102035. 34713634 10.1002/advs.202102035 PMC8655172

[33] Zhai Z , Qu X, Li H, Yang K, Wan P, Tan L, Ouyang Z, Liu X, Tian B, Xiao F, Wang W, Jiang C, Tang T, Fan Q, Qin A, Dai K. The effect of metallic magnesium degradation products on osteoclast-induced osteolysis and attenuation of NF-κB and NFATc1 signaling. Biomaterials 2014;35:6299–310. 24816285 10.1016/j.biomaterials.2014.04.044

[34] Wang L , Pang Y, Tang Y, Wang X, Zhang D, Zhang X, Yu Y, Yang X, Cai Q. A biomimetic piezoelectric scaffold with sustained Mg(2+) release promotes neurogenic and angiogenic differentiation for enhanced bone regeneration. Bioact Mater 2023;25:399–414. 37056250 10.1016/j.bioactmat.2022.11.004 PMC10087109

[35] Lin Z , Shen D, Zhou W, Zheng Y, Kong T, Liu X, Wu S, Chu PK, Zhao Y, Wu J, Cheung KMC, Yeung KWK. Regulation of extracellular bioactive cations in bone tissue microenvironment induces favorable osteoimmune conditions to accelerate in situ bone regeneration. Bioact Mater 2021;6:2315–30. 33553818 10.1016/j.bioactmat.2021.01.018 PMC7840811

[36] Tan S , Wang Y, Du Y, Xiao Y, Zhang S. Injectable bone cement with magnesium-containing microspheres enhances osteogenesis via anti-inflammatory immunoregulation. Bioact Mater 2021;6:3411–23. 33842737 10.1016/j.bioactmat.2021.03.006 PMC8010581

[37] Luo J , Zhu Y, Yu Y, Chen Y, He K, Liu J. Sinomenine treats rheumatoid arthritis by inhibiting MMP9 and inflammatory cytokines expression: bioinformatics analysis and experimental validation. Sci Rep 2024;14:12786. 38834626 10.1038/s41598-024-61769-x PMC11151427

[38] Yamaguchi T , Movila A, Kataoka S, Wisitrasameewong W, Ruiz Torruella M, Murakoshi M, Murakami S, Kawai T. Proinflammatory M1 macrophages inhibit RANKL-induced osteoclastogenesis. Infect Immun 2016;84:2802–12. 27456834 10.1128/IAI.00461-16 PMC5038061

[39] Nakazaki M , Morita T, Lankford KL, Askenase PW, Kocsis JD. Small extracellular vesicles released by infused mesenchymal stromal cells target M2 macrophages and promote TGF-β upregulation, microvascular stabilization and functional recovery in a rodent model of severe spinal cord injury. J Extracell Vesicles 2021;10:e12137. 34478241 10.1002/jev2.12137 PMC8408371

[40] Wang L , He C. Nrf2-mediated anti-inflammatory polarization of macrophages as therapeutic targets for osteoarthritis. Front Immunol 2022;13:967193. 36032081 10.3389/fimmu.2022.967193 PMC9411667

[41] Ono T , Nakashima T. Recent advances in osteoclast biology. Histochem Cell Biol 2018;149:325–41. 29392395 10.1007/s00418-018-1636-2

[42] Liang L , Song D, Wu K, Ouyang Z, Huang Q, Lei G, Zhou K, Xiao J, Wu H. Sequential activation of M1 and M2 phenotypes in macrophages by Mg degradation from Ti–Mg alloy for enhanced osteogenesis. Biomater Res 2022;26:17. 35484564 10.1186/s40824-022-00262-w PMC9052665

[43] Liang L , Yin Y, Guo Z, Liu T, Ouyang Z, Zhou J, Xiao J, Zhao L, Wu H. Sequentially activating macrophages M1 and M2 phenotypes by lipopolysaccharide-containing Mg–Fe layered double hydroxides coating on the Ti substrate. Colloids Surf B Biointerfaces 2023;222:113066. 36525754 10.1016/j.colsurfb.2022.113066

[44] Belluci MM , Schoenmaker T, Rossa-Junior C, Orrico SR, de Vries TJ, Everts V. Magnesium deficiency results in an increased formation of osteoclasts. J Nutr Biochem 2013;24:1488–98. 23517915 10.1016/j.jnutbio.2012.12.008

[45] Qin H , Zhao Y, An Z, Cheng M, Wang Q, Cheng T, Wang Q, Wang J, Jiang Y, Zhang X, Yuan G. Enhanced antibacterial properties, biocompatibility, and corrosion resistance of degradable Mg–Nd–Zn–Zr alloy. Biomaterials 2015;53:211–20. 25890720 10.1016/j.biomaterials.2015.02.096

[46] Andrés NC , Sieben JM, Baldini M, Rodríguez CH, Famiglietti Á, Messina PV. Electroactive Mg(2+)-hydroxyapatite nanostructured networks against drug-resistant bone infection strains. ACS Appl Mater Interfaces 2018;10:19534–44. 29799727 10.1021/acsami.8b06055

[47] Shibutani T , Heersche JN. Effect of medium pH on osteoclast activity and osteoclast formation in cultures of dispersed rabbit osteoclasts. J Bone Miner Res 1993;8:331–6. 7681246 10.1002/jbmr.5650080310

[48] Meghji S , Morrison MS, Henderson B, Arnett TR. pH dependence of bone resorption: mouse calvarial osteoclasts are activated by acidosis. Am J Physiol Endocrinol Metab 2001;280:E112–9. 11120665 10.1152/ajpendo.2001.280.1.E112

[49] Arnett TR. Extracellular pH regulates bone cell function. J Nutr 2008;138:415S–8S. 18203913 10.1093/jn/138.2.415S

[50] Goldhaber P , Rabadjija L. H+ stimulation of cell-mediated bone resorption in tissue culture. Am J Physiol 1987;253:E90–8. 3605336 10.1152/ajpendo.1987.253.1.E90

[51] Arnett TR , Spowage M. Modulation of the resorptive activity of rat osteoclasts by small changes in extracellular pH near the physiological range. Bone 1996;18:277–9. 8703584 10.1016/8756-3282(95)00486-6

[52] Gasser J , Ludwig M, Brandaoburch A, Ingold P, Ritter V, Arnett T, Seuwen K. Reduced BMD in mice lacking the pH-sensing receptor TDAG8 but not OGR1. Amer Soc Bone & Mineral Res 2006.

[53] Hoebertz A , Meghji S, Burnstock G, Arnett TR. Extracellular ADP is a powerful osteolytic agent: evidence for signaling through the P2Y(1) receptor on bone cells. FASEB J 2001;15:1139–48. 11344082 10.1096/fj.00-0395com

[54] Biskobing DM , Fan D. Acid pH increases carbonic anhydrase II and calcitonin receptor mRNA expression in mature osteoclasts. Calcif Tissue Int 2000;67:178–83. 10920224 10.1007/s00223001107

[55] Nordström T , Shrode LD, Rotstein OD, Romanek R, Goto T, Heersche JN, Manolson MF, Brisseau GF, Grinstein S. Chronic extracellular acidosis induces plasmalemmal vacuolar type H+ ATPase activity in osteoclasts. J Biol Chem 1997;272:6354–60. 9045656 10.1074/jbc.272.10.6354

[56] Brandao-Burch A , Meghji S, Arnett T. Acidosis strongly upregulates mRNA for cathepsin K, TRAP and TRAF-6 in bone. Calcif Tissue Int 2003;72:364.

[57] Hwang MA , Won M, Im JY, Kang MJ, Kweon DH, Kim BK. TNF-α secreted from macrophages increases the expression of prometastatic integrin αV in gastric cancer. Int J Mol Sci 2022;24:376–87. 36613819 10.3390/ijms24010376 PMC9820470

[58] Lagos-Cabré R , Alvarez A, Kong M, Burgos-Bravo F, Cárdenas A, Rojas-Mancilla E, Pérez-Nuñez R, Herrera-Molina R, Rojas F, Schneider P, Herrera-Marschitz M, Quest AFG, van Zundert B, Leyton L. αVβ_3_ integrin regulates astrocyte reactivity. J Neuroinflammation 2017;14:194. 28962574 10.1186/s12974-017-0968-5 PMC5622429

[59] Maradze D , Musson D, Zheng Y, Cornish J, Lewis M, Liu Y. High magnesium corrosion rate has an effect on osteoclast and mesenchymal stem cell role during bone remodelling. Sci Rep 2018;8:10003. 29968794 10.1038/s41598-018-28476-w PMC6030161

[60] Vignery A. Osteoclasts and giant cells: macrophage-macrophage fusion mechanism. Int J Exp Pathol 2000;81:291–304. 11168677 10.1111/j.1365-2613.2000.00164.x PMC2517739

[61] Honma M , Ikebuchi Y, Kariya Y, Suzuki H. Regulatory mechanisms of RANKL presentation to osteoclast precursors. Curr Osteoporos Rep 2014;12:115–20. 24477414 10.1007/s11914-014-0189-0

[62] Liu JZ , Ji ZL, Chen SM. [The OPG/RANKL/RANK system and bone resorptive disease]. Sheng Wu Gong Cheng Xue Bao 2003;19:655–60. 15971575

[63] Park JH , Lee NK, Lee SY. Current understanding of RANK signaling in osteoclast differentiation and maturation. Mol Cells 2017;40:706–13. 29047262 10.14348/molcells.2017.0225 PMC5682248

[64] Yao Z , Getting SJ, Locke IC. Regulation of TNF-induced osteoclast differentiation. Cells 2021;11:132. 35011694 10.3390/cells11010132 PMC8750957

[65] van Tuyl LH , Voskuyl AE, Boers M, Geusens P, Landewé RB, Dijkmans BA, Lems WF. Baseline RANKL: OPG ratio and markers of bone and cartilage degradation predict annual radiological progression over 11 years in rheumatoid arthritis. Ann Rheum Dis 2010;69:1623–8. 20525836 10.1136/ard.2009.121764

[66] Sugatani T , Hruska KA. Akt1/Akt2 and mammalian target of rapamycin/Bim play critical roles in osteoclast differentiation and survival, respectively, whereas Akt is dispensable for cell survival in isolated osteoclast precursors. J Biol Chem 2005;280:3583–9. 15545269 10.1074/jbc.M410480200

[67] Glantschnig H , Fisher JE, Wesolowski G, Rodan GA, Reszka AA. M-CSF, TNFalpha and RANK ligand promote osteoclast survival by signaling through mTOR/S6 kinase. Cell Death Differ 2003;10:1165–77. 14502240 10.1038/sj.cdd.4401285

[68] Darnay BG , Haridas V, Ni J, Moore PA, Aggarwal BB. Characterization of the intracellular domain of receptor activator of NF-kappaB (RANK). Interaction with tumor necrosis factor receptor-associated factors and activation of NF-kappab and c-jun N-terminal kinase. J Biol Chem 1998;273:20551–5. 9685412 10.1074/jbc.273.32.20551

[69] Kobayashi N , Kadono Y, Naito A, Matsumoto K, Yamamoto T, Tanaka S, Inoue J. Segregation of TRAF6-mediated signaling pathways clarifies its role in osteoclastogenesis. EMBO J 2001;20:1271–80. 11250893 10.1093/emboj/20.6.1271 PMC145527

[70] Frick KK , Bushinsky DA. Metabolic acidosis stimulates RANKL RNA expression in bone through a cyclo-oxygenase-dependent mechanism. J Bone Miner Res 2003;18:1317–25. 12854843 10.1359/jbmr.2003.18.7.1317

[71] Wada T , Nakashima T, Hiroshi N, Penninger JM. RANKL-RANK signaling in osteoclastogenesis and bone disease. Trends Mol Med 2006;12:17–25. 16356770 10.1016/j.molmed.2005.11.007

[72] Jin L , Chen C, Li Y, Yuan F, Gong R, Wu J, Zhang H, Kang B, Yuan G, Zeng H, Chen T. A biodegradable Mg-based alloy inhibited the inflammatory response of THP-1 cell-derived macrophages through the TRPM7-PI3K-AKT1 signaling axis. Front Immunol 2019;10:2798. 31849975 10.3389/fimmu.2019.02798 PMC6902094

[73] Zheng LZ , Wang JL, Xu JK, Zhang XT, Liu BY, Huang L, Zhang R, Zu HY, He X, Mi J, Pang QQ, Wang XL, Ruan YC, Zhao DW, Qin L. Magnesium and vitamin C supplementation attenuates steroid-associated osteonecrosis in a rat model. Biomaterials 2020;238:119828. 32045781 10.1016/j.biomaterials.2020.119828 PMC7185815

[74] Arancibia-Hernández YL , Aranda-Rivera AK, Cruz-Gregorio A, Pedraza-Chaverri J. Antioxidant/anti-inflammatory effect of Mg(2+) in coronavirus disease 2019 (COVID-19). Rev Med Virol 2022;32:e2348. 35357063 10.1002/rmv.2348 PMC9111052

[75] Rude RK , Gruber HE, Norton HJ, Wei LY, Frausto A, Mills BG. Bone loss induced by dietary magnesium reduction to 10% of the nutrient requirement in rats is associated with increased release of substance P and tumor necrosis factor-alpha. J Nutr 2004;134:79–85. 14704297 10.1093/jn/134.1.79

[76] Bae YJ , Kim MH. Calcium and magnesium supplementation improves serum OPG/RANKL in calcium-deficient ovariectomized rats. Calcif Tissue Int 2010;87:365–72. 20811796 10.1007/s00223-010-9410-z

[77] Sharif K , Sharif A, Jumah F, Oskouian R, Tubbs RS. Rheumatoid arthritis in review: clinical, anatomical, cellular and molecular points of view. Clin Anat 2018;31:216–23. 28833647 10.1002/ca.22980

[78] Cheng P , Weng Z, Hamushan M, Cai W, Zhang Y, Ren Z, Sun Y, Zhang X, Shen H, Han P. High-purity magnesium screws modulate macrophage polarization during the tendon-bone healing process in the anterior cruciate ligament reconstruction rabbit model. Regen Biomater 2022;9:rbac067. 36284747 10.1093/rb/rbac067 PMC9580517

[79] Zhao T , Chu Z, Chu CH, Dong S, Li G, Wu J, Tang C. Macrophages induce gingival destruction via Piezo1-mediated MMPs-degrading collagens in periodontitis. Front Immunol 2023;14:1194662. 37261355 10.3389/fimmu.2023.1194662 PMC10228731

[80] Wang X , Li Y, Feng Y, Cheng H, Li D. Macrophage polarization in aseptic bone resorption around dental implants induced by Ti particles in a murine model. J Periodontal Res 2019;54:329–38. 30635919 10.1111/jre.12633

[81] Vannella KM , Wynn TA. Mechanisms of organ injury and repair by macrophages. Annu Rev Physiol 2017;79:593–617. 27959618 10.1146/annurev-physiol-022516-034356

[82] Gao X , Ge J, Zhou W, Xu L, Geng D. IL-10 inhibits osteoclast differentiation and osteolysis through MEG3/IRF8 pathway. Cell Signal 2022;95:110353. 35525407 10.1016/j.cellsig.2022.110353

[83] Jiang J , Chen Q, Chen X, Li J, Li S, Yang B. Magnesium sulfate ameliorates sepsis-induced diaphragm dysfunction in rats via inhibiting HMGB1/TLR4/NF-κB pathway. Neuroreport 2020;31:902–8. 32558672 10.1097/WNR.0000000000001478 PMC7368847

[84] Heinecke JW , Rosen H, Suzuki LA, Chait A. The role of sulfur-containing amino acids in superoxide production and modification of low density lipoprotein by arterial smooth muscle cells. J Biol Chem 1987;262:10098–103. 3038867

[85] Rubin H. The membrane, magnesium, mitosis (MMM) model of cell proliferation control. Magnes Res 2005;18:268–74. 16548142

[86] Nartea R , Mitoiu BI, Ghiorghiu I. The link between magnesium supplements and statin medication in dyslipidemic patients. Curr Issues Mol Biol 2023;45:3146–67. 37185729 10.3390/cimb45040205 PMC10136538

[87] Sun L , Li X, Xu M, Yang F, Wang W, Niu X. In vitro immunomodulation of magnesium on monocytic cell toward anti-inflammatory macrophages. Regen Biomater 2020;7:391–401. 32793384 10.1093/rb/rbaa010 PMC7415003

[88] Liang L , Lin Z, Duan Z, Agbedor SO, Li N, Baker I, Wang B, Liu T, Wu H. Enhancing the immunomodulatory osteogenic properties of Ti–Mg alloy by Mg(2+)-containing nanostructures. Regen Biomater 2024;11:rbae104. 39372848 10.1093/rb/rbae104 PMC11453102

[89] Hadjidakis DJ , Androulakis II. Bone remodeling. Ann N Y Acad Sci 2006;1092:385–96. 17308163 10.1196/annals.1365.035

[90] Lutter AH , Hempel U, Anderer U, Dieter P. Biphasic influence of PGE2 on the resorption activity of osteoclast-like cells derived from human peripheral blood monocytes and mouse RAW264.7 cells. Prostaglandins Leukot Essent Fatty Acids 2016;111:1–7. 27499447 10.1016/j.plefa.2016.03.017

[91] Pilbeam C. Prostaglandins and bone. Handb Exp Pharmacol 2020;262:157–75. 31820176 10.1007/164_2019_332

[92] Tahara H , Nishizawa Y. [Hypomagnesemia and hypoparathyroidism]. Clin Calcium 2007;17:1200–4. 17660616

[93] Rude RK , Gruber HE, Norton HJ, Wei LY, Frausto A, Kilburn J. Reduction of dietary magnesium by only 50% in the rat disrupts bone and mineral metabolism. Osteoporos Int 2006;17:1022–32. 16601920 10.1007/s00198-006-0104-3

[94] Rosanoff A , Seelig MS. Comparison of mechanism and functional effects of magnesium and statin pharmaceuticals. J Am Coll Nutr 2004;23:501s–5s. 15466951 10.1080/07315724.2004.10719389

[95] Xu Y , Lv C, Zhang J, Li Y, Li T, Zhang C, Chen J, Bai D, Yin X, Zou S. Intermittent parathyroid hormone promotes cementogenesis in a PKA- and ERK1/2-dependent manner. J Periodontol 2019;90:1002–13. 31026057 10.1002/JPER.18-0639

[96] Chen YJ , Wang SP, Cheng FC, Hsu PY, Li YF, Wu J, Huang HL, Tsai MT, Hsu JT. Intermittent parathyroid hormone improve bone microarchitecture of the mandible and femoral head in ovariectomized rats. BMC Musculoskelet Disord 2017;18:171. 28438150 10.1186/s12891-017-1530-4 PMC5404672

[97] Li JY , Yu M, Tyagi AM, Vaccaro C, Hsu E, Adams J, Bellido T, Weitzmann MN, Pacifici R. IL-17 receptor signaling in osteoblasts/osteocytes mediates PTH-induced bone loss and enhances osteocytic RANKL production. J Bone Miner Res 2019;34:349–60. 30399207 10.1002/jbmr.3600

[98] Ricarte FR , Le Henaff C, Kolupaeva VG, Gardella TJ, Partridge NC. Parathyroid hormone(1–34) and its analogs differentially modulate osteoblastic Rankl expression via PKA/SIK2/SIK3 and PP1/PP2A-CRTC3 signaling. J Biol Chem 2018;293:20200–13. 30377251 10.1074/jbc.RA118.004751 PMC6311504

[99] Sun P , Wang M, Yin GY. Endogenous parathyroid hormone (PTH) signals through osteoblasts via RANKL during fracture healing to affect osteoclasts. Biochem Biophys Res Commun 2020;525:850–6. 32169280 10.1016/j.bbrc.2020.02.177

[100] Wein MN , Liang Y, Goransson O, Sundberg TB, Wang J, Williams EA, O'Meara MJ, Govea N, Beqo B, Nishimori S, Nagano K, Brooks DJ, Martins JS, Corbin B, Anselmo A, Sadreyev R, Wu JY, Sakamoto K, Foretz M, Xavier RJ, Baron R, Bouxsein ML, Gardella TJ, Divieti-Pajevic P, Gray NS, Kronenberg HM. SIKs control osteocyte responses to parathyroid hormone. Nat Commun 2016;7:13176. 27759007 10.1038/ncomms13176 PMC5075806

[101] Aydin H , Deyneli O, Yavuz D, Gözü H, Mutlu N, Kaygusuz I, Akalin S. Short-term oral magnesium supplementation suppresses bone turnover in postmenopausal osteoporotic women. Biol Trace Elem Res 2010;133:136–43. 19488681 10.1007/s12011-009-8416-8

[102] Kobayashi Y , Mizoguchi T, Take I, Kurihara S, Udagawa N, Takahashi N. Prostaglandin E2 enhances osteoclastic differentiation of precursor cells through protein kinase A-dependent phosphorylation of TAK1. J Biol Chem 2005;280:11395–403. 15647289 10.1074/jbc.M411189200

[103] Hackett JA , Allard-Chamard H, Sarrazin P, de Fatima Lucena M, Gallant MA, Fortier I, Nader M, Parent JL, Bkaily G, de Brum-Fernandes AJ. Prostaglandin production by human osteoclasts in culture. J Rheumatol 2006;33:1320–8. 16758505

[104] Okamoto F , Kajiya H, Fukushima H, Jimi E, Okabe K. Prostaglandin E2 activates outwardly rectifying Cl(−) channels via a cAMP-dependent pathway and reduces cell motility in rat osteoclasts. Am J Physiol Cell Physiol 2004;287:C114–24. 15044156 10.1152/ajpcell.00551.2003

[105] Owan I , Ibaraki K. The role of calcitonin gene-related peptide (CGRP) in macrophages: the presence of functional receptors and effects on proliferation and differentiation into osteoclast-like cells. Bone Miner 1994;24:151–64. 8199534 10.1016/s0169-6009(08)80152-3

[106] Naot D , Cornish J. The role of peptides and receptors of the calcitonin family in the regulation of bone metabolism. Bone 2008;43:813–8. 18687416 10.1016/j.bone.2008.07.003

[107] Alam AS , Moonga BS, Bevis PJ, Huang CL, Zaidi M. Amylin inhibits bone resorption by a direct effect on the motility of rat osteoclasts. Exp Physiol 1993;78:183–96. 8385961 10.1113/expphysiol.1993.sp003679

[108] Ohsawa I , Ishikawa M, Takahashi K, Watanabe M, Nishimaki K, Yamagata K, Katsura K, Katayama Y, Asoh S, Ohta S. Hydrogen acts as a therapeutic antioxidant by selectively reducing cytotoxic oxygen radicals. Nat Med 2007;13:688–94. 17486089 10.1038/nm1577

[109] Zhao D , Brown A, Wang T, Yoshizawa S, Sfeir C, Heineman WR. In vivo quantification of hydrogen gas concentration in bone marrow surrounding magnesium fracture fixation hardware using an electrochemical hydrogen gas sensor. Acta Biomater 2018;73:559–66. 29684620 10.1016/j.actbio.2018.04.032

[110] Qin ZX , Yu P, Qian DH, Song MB, Tan H, Yu Y, Li W, Wang H, Liu J, Wang Q, Sun XJ, Jiang H, Zhu JK, Lu W, Huang L. Hydrogen-rich saline prevents neointima formation after carotid balloon injury by suppressing ROS and the TNF-α/NF-κB pathway. Atherosclerosis 2012;220:343–50. 22153150 10.1016/j.atherosclerosis.2011.11.002

[111] Chen S , Yu Y, Xie S, Liang D, Shi W, Chen S, Li G, Tang W, Liu C, He Q. Local H(2) release remodels senescence microenvironment for improved repair of injured bone. Nat Commun 2023;14:7783. 38012166 10.1038/s41467-023-43618-z PMC10682449

[112] Srinivasan S , Koenigstein A, Joseph J, Sun L, Kalyanaraman B, Zaidi M, Avadhani NG. Role of mitochondrial reactive oxygen species in osteoclast differentiation. Ann N Y Acad Sci 2010;1192:245–52. 20392243 10.1111/j.1749-6632.2009.05377.x PMC2856121

[113] Agidigbi TS , Kim C. Reactive oxygen species in osteoclast differentiation and possible pharmaceutical targets of ROS-mediated osteoclast diseases. Int J Mol Sci 2019;20:3576–91. 31336616 10.3390/ijms20143576 PMC6678498

[114] Schröder K. NADPH oxidases in bone homeostasis and osteoporosis. Free Radic Biol Med 2019;132:67–72. 30189265 10.1016/j.freeradbiomed.2018.08.036

[115] Bonaccorsi G , Piva I, Greco P, Cervellati C. Oxidative stress as a possible pathogenic cofactor of post-menopausal osteoporosis: existing evidence in support of the axis oestrogen deficiency-redox imbalance-bone loss. Indian J Med Res 2018;147:341–51. 29998869 10.4103/ijmr.IJMR_524_18 PMC6057254

[116] Ha H , Kwak HB, Lee SW, Jin HM, Kim HM, Kim HH, Lee ZH. Reactive oxygen species mediate RANK signaling in osteoclasts. Exp Cell Res 2004;301:119–27. 15530848 10.1016/j.yexcr.2004.07.035

[117] Li DZ , Zhang QX, Dong XX, Li HD, Ma X. Treatment with hydrogen molecules prevents RANKL-induced osteoclast differentiation associated with inhibition of ROS formation and inactivation of MAPK, AKT and NF-kappa B pathways in murine RAW264.7 cells. J Bone Miner Metab 2014;32:494–504. 24196871 10.1007/s00774-013-0530-1

[118] Fang W , Wang G, Tang L, Su H, Chen H, Liao W, Xu J. Hydrogen gas inhalation protects against cutaneous ischaemia/reperfusion injury in a mouse model of pressure ulcer. J Cell Mol Med 2018;22:4243–52. 29921037 10.1111/jcmm.13704 PMC6111801

[119] Zhang B , Zhao Z, Meng X, Chen H, Fu G, Xie K. Hydrogen ameliorates oxidative stress via PI3K-Akt signaling pathway in UVB-induced HaCaT cells. Int J Mol Med 2018;41:3653–61. 29532858 10.3892/ijmm.2018.3550

[120] Ji P , Qiu S, Huang J, Wang L, Wang Y, Wu P, Huo M, Shi J. Hydrolysis of 2D nanosheets reverses rheumatoid arthritis through anti-inflammation and osteogenesis. Adv Mater 2025;37:e2415543. 39726077 10.1002/adma.202415543

[121] Zhou H , He Z, Cao Y, Chu L, Liang B, Yu K, Deng Z. An injectable magnesium-loaded hydrogel releases hydrogen to promote osteoporotic bone repair via ROS scavenging and immunomodulation. Theranostics 2024;14:3739–59. 38948054 10.7150/thno.97412 PMC11209720

[122] Guo SX , Fang Q, You CG, Jin YY, Wang XG, Hu XL, Han CM. Effects of hydrogen-rich saline on early acute kidney injury in severely burned rats by suppressing oxidative stress induced apoptosis and inflammation. J Transl Med 2015;13:183. 26047940 10.1186/s12967-015-0548-3 PMC4467622

[123] Hou YC , Witte F, Li J, Guan S. The increased ratio of Mg2+/Ca2+ from degrading magnesium alloys directs macrophage fate for functionalized growth of endothelial cells. Smart Mater Med 2022;3:188–98.

[124] Myrissa A , Agha NA, Lu Y, Martinelli E, Eichler J, Szakács G, Kleinhans C, Willumeit-Römer R, Schäfer U, Weinberg AM. In vitro and in vivo comparison of binary Mg alloys and pure Mg. Mater Sci Eng C Mater Biol Appl 2016;61:865–74. 26838918 10.1016/j.msec.2015.12.064

[125] Wang C , Song C, Mei D, Wang L, Wang W, Wu T, Snihirova D, Zheludkevich ML, Lamaka SV. Control i. Low interfacial pH discloses the favorable biodegradability of several Mg alloys. Corros Sci 2022;197:110059.

[126] Wang W , Blawert C, Zan R, Sun Y, Peng H, Ni J, Han P, Suo T, Song Y, Zhang S, Zheludkevich ML, Zhang X. A novel lean alloy of biodegradable Mg–2Zn with nanograins. Bioact Mater 2021;6:4333–41. 33997510 10.1016/j.bioactmat.2021.04.020 PMC8105637

[127] Yang Y , Lu C, Shen L, Zhao Z, Peng S, Shuai CJ. In-situ deposition of apatite layer to protect Mg-based composite fabricated via laser additive manufacturing. J Magnes Alloys 2023;11:629–40.

[128] Bakhsheshi-Rad HR , Ismail AF, Aziz M, Akbari M, Hadisi Z, Khoshnava SM, Pagan E, Chen X. Co-incorporation of graphene oxide/silver nanoparticle into poly-L-lactic acid fibrous: a route toward the development of cytocompatible and antibacterial coating layer on magnesium implants. Mater Sci Eng C Mater Biol Appl 2020;111:110812. 32279830 10.1016/j.msec.2020.110812

[129] Zhu Y , Zhou M, Zhao W, Geng Y, Chen Y, Tian H, Zhou Y, Chen G, Wu R, Zheng Y, Shi Q. Insight the long-term biodegradable Mg–RE–Sr alloy for orthopaedics implant via friction stir processing. Bioact Mater 2024;41:293–311. 39157692 10.1016/j.bioactmat.2024.07.021 PMC11327549

[130] Zhang C , Zhang S, Sun D, Lin J, HJJoM L. Superhydrophobic fluoride conversion coating on bioresorbable magnesium alloy—fabrication, characterization, degradation and cytocompatibility with BMSCs. J Magnes Alloy 2021;9:1246–60.

[131] Mao L , Yuan G, Niu J, Zong Y, Ding W. In vitro degradation behavior and biocompatibility of Mg–Nd–Zn–Zr alloy by hydrofluoric acid treatment. Mater Sci Eng C Mater Biol Appl 2013;33:242–50. 25428068 10.1016/j.msec.2012.08.036

[132] Iranshahi F , Nasiri MB, Warchomicka FG, Sommitsch CJ. Investigation of the degradation rate of electron beam processed and friction stir processed biocompatible ZKX50 magnesium alloy. J Magnes Alloys 2022;10:768–81.

[133] Chen H , Yuan B, Zhao R, Yang X, Xiao Z, Aurora A, Iulia BA, Zhu X, Iulian AV, Zhang XJ. Evaluation on the corrosion resistance, antibacterial property and osteogenic activity of biodegradable Mg–Ca and Mg–Ca–Zn–Ag alloys. J Magnes Alloys 2022;10:3380–96.

[134] Zou YH , Wang J, Cui LY, Zeng RC, Wang QZ, Han QX, Qiu J, Chen XB, Chen DC, Guan SK, Zheng YF. Corrosion resistance and antibacterial activity of zinc-loaded montmorillonite coatings on biodegradable magnesium alloy AZ31. Acta Biomater 2019;98:196–214. 31154057 10.1016/j.actbio.2019.05.069

[135] Miao H , Zhang D, Chen C, Zhang L, Pei J, Su Y, Huang H, Wang Z, Kang B, Ding W, Zeng H, Yuan G. Research on biodegradable Mg–Zn–Gd alloys for potential orthopedic implants: in vitro and in vivo evaluations. ACS Biomater Sci Eng 2019;5:1623–34. 33405635 10.1021/acsbiomaterials.8b01563

[136] Zhang Y , Cao J, Lu M, Shao Y, Jiang K, Yang X, Xiong X, Wang S, Chu C, Xue F, Ye Y, Bai J. A biodegradable magnesium surgical staple for colonic anastomosis: in vitro and in vivo evaluation. Bioact Mater 2023;22:225–38. 36254273 10.1016/j.bioactmat.2022.09.023 PMC9550537

[137] Lin S , Yang G, Jiang F, Zhou M, Yin S, Tang Y, Tang T, Zhang Z, Zhang W, Jiang X. A magnesium-enriched 3D culture system that mimics the bone development microenvironment for vascularized bone regeneration. Adv Sci (Weinh) 2019;6:1900209. 31380166 10.1002/advs.201900209 PMC6662069

[138] Yoshizawa S , Brown A, Barchowsky A, Sfeir C. Magnesium ion stimulation of bone marrow stromal cells enhances osteogenic activity, simulating the effect of magnesium alloy degradation. Acta Biomater 2014;10:2834–42. 24512978 10.1016/j.actbio.2014.02.002

[139] He W , Zhang H, Qiu J. Osteogenic effects of bioabsorbable magnesium implant in rat mandibles and in vitro. J Periodontol 2021;92:1181–91. 32846010 10.1002/JPER.20-0162

[140] Wang Y , Geng Z, Huang Y, Jia Z, Cui Z, Li Z, Wu S, Liang Y, Zhu S, Yang X, Lu WW. Unraveling the osteogenesis of magnesium by the activity of osteoblasts in vitro. J Mater Chem B 2018;6:6615–21. 32254870 10.1039/c8tb01746h

[141] Liu W , Guo S, Tang Z, Wei X, Gao P, Wang N, Li X, Guo Z. Magnesium promotes bone formation and angiogenesis by enhancing MC3T3-E1 secretion of PDGF-BB. Biochem Biophys Res Commun 2020;528:664–70. 32513539 10.1016/j.bbrc.2020.05.113

[142] Lin S , Yin S, Shi J, Yang G, Wen X, Zhang W, Zhou M, Jiang X. Orchestration of energy metabolism and osteogenesis by Mg(2+) facilitates low-dose BMP-2-driven regeneration. Bioact Mater 2022;18:116–27. 35387176 10.1016/j.bioactmat.2022.03.024 PMC8961427

[143] Bernhardt A , Helmholz H, Kilian D, Willumeit-Römer R, Gelinsky M. Impact of degradable magnesium implants on osteocytes in single and triple cultures. Biomater Adv 2022;134:112692. 35581081 10.1016/j.msec.2022.112692

[144] Della Rosa G , Gostynska NE, Ephraim JW, Sganga S, Panuccio G, Palazzolo G, Tirelli N. Magnesium alginate as a low-viscosity (intramolecularly cross-linked) system for the sustained and neuroprotective release of magnesium. Carbohydr Polym 2024;331:121871. 38388038 10.1016/j.carbpol.2024.121871

[145] Zhang Y , Zhang H, Jiang M, Cao X, Ge X, Song B, Lan J, Zhou W, Qi Z, Gu X, Liu J, Zheng Y, Li M, Ji X. Neuroprotection on ischemic brain injury by Mg(2+)/H(2) released from endovascular Mg implant. Bioact Mater 2024;42:124–39. 39280580 10.1016/j.bioactmat.2024.08.019 PMC11402188

[146] Nie X , Sun X, Wang C, Yang J. Effect of magnesium ions/type I collagen promote the biological behavior of osteoblasts and its mechanism. Regen Biomater 2020;7:53–61. 32440359 10.1093/rb/rbz033 PMC7233620

[147] Amerstorfer F , Fischerauer SF, Fischer L, Eichler J, Draxler J, Zitek A, Meischel M, Martinelli E, Kraus T, Hann S, Stanzl-Tschegg SE, Uggowitzer PJ, Löffler JF, Weinberg AM, Prohaska T. Long-term in vivo degradation behavior and near-implant distribution of resorbed elements for magnesium alloys WZ21 and ZX50. Acta Biomater 2016;42:440–50. 27343708 10.1016/j.actbio.2016.06.025

[148] Zhao D , Wang T, Kuhlmann J, Dong Z, Chen S, Joshi M, Salunke P, Shanov VN, Hong D, Kumta PN, Heineman WR. In vivo monitoring the biodegradation of magnesium alloys with an electrochemical H2 sensor. Acta Biomater 2016;36:361–8. 27045693 10.1016/j.actbio.2016.03.039

[149] Marek R , Ćwieka H, Donohue N, Holweg P, Moosmann J, Beckmann F, Brcic I, Schwarze UY, Iskhakova K, Chaabane M, Sefa S, Zeller-Plumhoff B, Weinberg AM, Willumeit-Römer R, Sommer NG. Degradation behavior and osseointegration of Mg–Zn–Ca screws in different bone regions of growing sheep: a pilot study. Regen Biomater 2023;10:rbac077. 36683753 10.1093/rb/rbac077 PMC9845522

[150] Badkoobeh F , Mostaan H, Rafiei M, Bakhsheshi‐Rad HR, Ramakrishna S, XJJoM C. Alloys. Additive manufacturing of biodegradable magnesium-based materials: design strategies, properties, and biomedical applications. J Magnes Alloy 2023;11:801–39.

[151] Yin S , Lin S, Xu J, Yang G, Chen H, Jiang X. Dominoes with interlocking consequences triggered by zinc: involvement of microelement-stimulated MSC-derived exosomes in senile osteogenesis and osteoclast dialogue. J Nanobiotechnology 2023;21:346. 37741978 10.1186/s12951-023-02085-w PMC10518091

[152] Lee NH , Kang MS, Kim TH, Yoon DS, Mandakhbayar N, Jo SB, Kim HS, Knowles JC, Lee JH, Kim HW. Dual actions of osteoclastic-inhibition and osteogenic-stimulation through strontium-releasing bioactive nanoscale cement imply biomaterial-enabled osteoporosis therapy. Biomaterials 2021;276:121025. 34298444 10.1016/j.biomaterials.2021.121025

[153] Zhang S , Sun X, Kang C, Yang M, Zhao Y, Wang C. Study on repairing canine mandibular defect with porous Mg–Sr alloy combined with Mg–Sr alloy membrane. Regen Biomater 2020;7:331–6. 32523734 10.1093/rb/rbz046 PMC7266669

[154] Li JX , Chen JX, Siddiqui MA, Kolawole SK, Yang Y, Shen Y, Yang JP, Wang JH, Su XPJ. Enhancing corrosion resistance and antibacterial properties of ZK60 magnesium alloy using micro-arc oxidation coating containing nano-zinc oxide. Acta Metall Sin (Engl Lett) 2025;38:45–58.

[155] Nie T , Fang Y, Zhang R, Cai Y, Wang X, Jiao Y, Wu J. Self-healable and pH-responsive spermidine/ferrous ion complexed hydrogel Co-loaded with CA inhibitor and glucose oxidase for combined cancer immunotherapy through triple ferroptosis mechanism. Bioact Mater 2025;47:51–63. 39877156 10.1016/j.bioactmat.2025.01.005 PMC11772096

[156] Zhao Y , He P, Yao J, Li M, Wang B, Han L, Huang Z, Guo C, Bai J, Xue F, Cong Y, Cai W, Chu PK, Chu C. pH/NIR-responsive and self-healing coatings with bacteria killing, osteogenesis, and angiogenesis performances on magnesium alloy. Biomaterials 2023;301:122237. 37467596 10.1016/j.biomaterials.2023.122237

[157] Shao H , Cheng S, Yao M, Ji X, Zhong H, Wang D, Fan X, Li Q, Zhou J, Zhang Y, Peng F. A pH-response chemotherapy synergistic photothermal therapy for tumor suppression and bone regeneration by mussel-inspired Mg implant. Regen Biomater 2021;8:rbab053. 34557310 10.1093/rb/rbab053 PMC8455343

[158] Vinikoor T , Dzidotor GK, Le TT, Liu Y, Kan HM, Barui S, Chorsi MT, Curry EJ, Reinhardt E, Wang H, Singh P, Merriman MA, D'Orio E, Park J, Xiao S, Chapman JH, Lin F, Truong CS, Prasadh S, Chuba L, Killoh S, Lee SW, Wu Q, Chidambaram RM, Lo KWH, Laurencin CT, Nguyen TD. Injectable and biodegradable piezoelectric hydrogel for osteoarthritis treatment. Nat Commun 2023;14:6257. 37802985 10.1038/s41467-023-41594-y PMC10558537

[159] Dubey A , Jaiswal S, Haldar S, Roy P, Lahiri D. Functionally gradient magnesium-based composite for temporary orthopaedic implant with improved corrosion resistance and osteogenic properties. Biomed Mater 2020;16:015017. 33325376 10.1088/1748-605X/abb721

[160] Zhang W , Lu J, Tan L, Ni D, Zhang R, Zhou Q, Yang K, Wang Q. In vitro degradation and in vivo osteogenesis of Mg–Zn–Nd–Zr/HA composites prepared by friction stir processing. J Magnes Alloys 2024;12:4937–52.

[161] Zheng Q , Li J, Yuan W, Liu X, Wu S. Engineering. Metal–organic frameworks incorporated polycaprolactone film for enhanced corrosion resistance and biocompatibility of Mg alloy. ACS Sustain Chem Eng 2019;7:18114–24.

[162] Li W , Huang Y, Gu D, Peng S, Zhang B, Peng F, Zhang D, Li M, Xiao J, Jia Z, Qiu L. Ascorbate-loaded MgFe layered double hydroxide for osteomyelitis treatment. J Control Release 2025;378:1045–60. 39740696 10.1016/j.jconrel.2024.12.072

[163] Huang S , Li J, Qin K, Wang Z, Yang J, Cao F, Li W, Liu Y, Liu L, Zhao D. Evaluation of the performance of Ca-deficient hydroxyapatite (CDHA)/MgF(2) bilayer coating on biodegradable high-purity magnesium in a femoral condyle defect model in rabbits. Regen Biomater 2022;9:rbac066. 36226163 10.1093/rb/rbac066 PMC9550228

[164] Lin Z , Wu J, Qiao W, Zhao Y, Wong KHM, Chu PK, Bian L, Wu S, Zheng Y, Cheung KMC, Leung F, Yeung KWK. Precisely controlled delivery of magnesium ions thru sponge-like monodisperse PLGA/nano-MgO-alginate core–shell microsphere device to enable in-situ bone regeneration. Biomaterials 2018;174:1–16. 29763774 10.1016/j.biomaterials.2018.05.011

[165] Wang P , Wu J, Yang H, Liu H, Yao T, Liu C, Gong Y, Wang M, Ji G, Huang P, Wang X. Intelligent microneedle patch with prolonged local release of hydrogen and magnesium ions for diabetic wound healing. Bioact Mater 2023;24:463–76. 36685806 10.1016/j.bioactmat.2023.01.001 PMC9841127

[166] Swain S , Patra A, Kumari S, Praharaj R, Mishra S, Rautray T. Corona poled gelatin—magnesium hydroxyapatite composite demonstrates osteogenicity. Mater Today Proc 2022;62:5.

[167] Guangyi L , Minfang C, Chen Y, Zhen L, Yun W, Wei LJMC. Preparation and characterization of biodegradable Mg–Zn–Ca/MgO nanocomposites for biomedical applications. Mater Character 2018;144:120–30.

[168] Zhou C , Liang G, Liu Y, Zhang H, Chen X, Li Y. Pore structure of porous Mg-1Mn-xZn alloy fabricated by metal–gas eutectic unidirectional solidification. J Magnes Alloys 2022;10:2137–46.

[169] Chen W , Sheng S, Tan K, Wang S, Wu X, Yang J, Hu Y, Cao L, Xu K, Zhou F, Su J, Zhang Q, Yang L. Injectable hydrogels for bone regeneration with tunable degradability via peptide chirality modification. Mater Horiz 2024;11:4367–77. 38932613 10.1039/d4mh00398e

[170] Liao J , Zhang J, Li J, Zeng Y, Dai Y, Xiao T, Xia Y, Li Y, Li D, Zhang DA. Hydrogel containing Mg^2+^ with improved osteogenesis, enhanced endochondral ossification, and modulated inflammation for bone-repair applications. Chem Eng J 2024;493:152762.

[171] Qi L , Fang X, Yan J, Pan C, Ge W, Wang J, Shen SG, Lin K, Zhang L. Magnesium-containing bioceramics stimulate exosomal miR-196a-5p secretion to promote senescent osteogenesis through targeting Hoxa7/MAPK signaling axis. Bioact Mater 2024;33:14–29. 38024235 10.1016/j.bioactmat.2023.10.024 PMC10661166

[172] Yang N , Yang X, Cheng S, Gao X, Sun S, Huang X, Ge J, Han Z, Huang C, Wang Y, Cheng C, Cheng L. Magnesium implants with alternating magnetic field-enhanced hydrogen release and proton depletion for anti-infection treatment and tissue repair. Bioact Mater 2024;38:374–83. 38770429 10.1016/j.bioactmat.2024.05.010 PMC11103218

[173] Golafshan N , Vorndran E, Zaharievski S, Brommer H, Kadumudi FB, Dolatshahi-Pirouz A, Gbureck U, van Weeren R, Castilho M, Malda J. Tough magnesium phosphate-based 3D-printed implants induce bone regeneration in an equine defect model. Biomaterials 2020;261:120302. 32932172 10.1016/j.biomaterials.2020.120302 PMC7116184

[174] Kang Y , Xu C, Meng L, Dong X, Qi M, Jiang D. Exosome-functionalized magnesium-organic framework-based scaffolds with osteogenic, angiogenic and anti-inflammatory properties for accelerated bone regeneration. Bioact Mater 2022;18:26–41. 35387167 10.1016/j.bioactmat.2022.02.012 PMC8961306

[175] Liu J , Liu B, Min S, Yin B, Peng B, Yu Z, Wang C, Ma X, Wen P, Tian Y, Zheng Y. Biodegradable magnesium alloy WE43 porous scaffolds fabricated by laser powder bed fusion for orthopedic applications: process optimization, in vitro and in vivo investigation. Bioact Mater 2022;16:301–19. 35415288 10.1016/j.bioactmat.2022.02.020 PMC8965912

[176] Yang X , Fan Y, Liang J, Cao R, Zhang B, Li J, Li Z, He S, Liu N, Du J, Hu Y. Polyaptamer-driven crystallization of alendronate for synergistic osteoporosis treatment through osteoclastic inhibition and osteogenic promotion. ACS Nano 2024;18:22431–43. 39103298 10.1021/acsnano.4c07265

[177] Zhang P , Wang T, Qian J, Qin H, Liu P, Xiong A, Udduttula A, Wang D, Zeng H, Chen Y. An injectable magnesium-coordinated phosphate chitosan-based hydrogel loaded with vancomycin for antibacterial and osteogenesis in the treatment of osteomyelitis. Regen Biomater 2024;11:rbae049. 38919844 10.1093/rb/rbae049 PMC11196881

[178] Lian C , Liu J, Wei W, Wu X, Goto T, Li H, Tu R, Dai H. Mg-gallate metal–organic framework-based sprayable hydrogel for continuously regulating oxidative stress microenvironment and promoting neurovascular network reconstruction in diabetic wounds. Bioact Mater 2024;38:181–94. 38711758 10.1016/j.bioactmat.2024.04.028 PMC11070761

[179] Guo J , Yao H, Chang L, Zhu W, Zhang Y, Li X, Yang B, Dai B, Chen X, Lei L, Chen Z, Li Y, Zheng L, Liu W, Tong W, Su Y, Qin L, Xu J. Magnesium nanocomposite hydrogel reverses the pathologies to enhance mandible regeneration. Adv Mater 2025;37:e2312920. 39385647 10.1002/adma.202312920 PMC11733717

[180] Wang N , Yang S, Shi H, Song Y, Sun H, Wang Q, Tan L, Guo SJ. Magnesium alloys for orthopedic applications: a review on the mechanisms driving bone healing. J Magnes Alloys 2022;10:3327–53.

